# The Sigma1 ER membrane receptor promotes structural protein folding and genome packaging of dengue virus

**DOI:** 10.1371/journal.ppat.1014347

**Published:** 2026-07-16

**Authors:** Riya Sarkar, Whitney Reid, Renaldo Sutanto, Andrew W. Tai, Billy Tsai

**Affiliations:** 1 Department of Cell and Developmental Biology, University of Michigan Medical School, Ann Arbor, Michigan, United States of America; 2 Cellular and Molecular Biology Program, University of Michigan Medical School, Ann Arbor, Michigan, United States of America; 3 Department of Internal Medicine, University of Michigan Medical School, Ann Arbor, Michigan, United States of America; 4 Department of Microbiology & Immunology, University of Michigan Medical School, Ann Arbor, Michigan, United States of America; Heidelberg University, GERMANY

## Abstract

Dengue virus (DENV) exploits the host endoplasmic reticulum (ER) to support viral protein translation and folding, replication, and assembly, although the identity of ER factors that promote these distinct steps during infection remain unclear. Here we demonstrate that the ER-resident Sigma1 ER membrane receptor (S1R) promotes virus structural protein folding and genome packaging of DENV during infection. Under S1R knockdown (KD), DENV infection is impaired without compromising virus translation or replication. Strikingly, EM analysis revealed that DENV particles in and secreted from S1R-depleted cells are smaller, likely because they are empty particles devoid of the vRNA genome. Biochemical experiments demonstrated that S1R binds to the prM structural protein and under S1R KD, the prM, E and C structural proteins became detergent-insoluble. Thus, without S1R, all three virus structural proteins misfold, impairing efficient genome packaging. Together, these findings identify a novel ER chaperone that supports a critical DENV infection step.

## Author summary

DENV exploits the host ER for viral protein translation and folding, replication, and assembly, although the specific host factors responsible for each step remains unclear. In this study, we identify S1R as an ER-resident host chaperone that promotes folding of DENV structural proteins to enable efficient genome packaging.

## Introduction

Dengue virus (DENV), which belongs to the Orthoflavivirus genus within the Flaviviridae family, is responsible for the most common human arboviral infection worldwide, accounting for about 390 million acute infections, 500,000 hospitalizations, and 25,000 deaths annually.[1] Despite this global health impact, the cellular infection mechanism of DENV is not entirely understood. The infection lifecycle of DENV begins when the virus undergoes endocytosis to reach the late endosome (LE).[2] Here the low pH triggers fusion of the virus and LE membranes, releasing the positive-strand virus RNA (vRNA) into the cytosol.[2,3] The vRNA is targeted to the endoplasmic reticulum (ER) where it undergoes translation, generating a single multi-pass transmembrane polyprotein that harbors—from the N- to the C-terminus—the three structural proteins capsid (C), premembrane (prM), envelope (E), along with the seven non-structural proteins NS1, NS2A, NS2B, NS3, NS4A, NS4B, and NS5.[3] The polyprotein then folds to its native conformation and is cleaved by viral and host proteases to form the individual proteins.[2,3]The nonstructural proteins support replication of vRNA in virus-induced ER-derived replication organelles containing individual vesicular packets (Vps).[4,5] The replicated vRNA, in complex with the C protein, assembles with the prM-E structural proteins at dilated ER cisternae that represent particle assembly sites (which are distinct from the replication organelles) to produce immature viral particles.[6,7] During ER-dependent assembly, the prM and E structural proteins interact directly with each other and oligomerize, inducing ER membrane invagination that buds the nascent viral particle into the ER lumen [7]. The vRNA-C protein complex is thought to be passively engulfed during the budding process to complete genome packaging. After ER-dependent assembly, immature virions exit the ER assembly site and then mature in the *trans* Golgi network (TGN) via furin-mediated cleavage of prM to form M [8], generating infectious viruses that are secreted from the host.[9]

Because virus protein translation, folding, replication, assembly, and genome packaging are distinct steps that all depend on the host ER, elucidating and uncoupling the mechanisms underlying these steps is challenging. Not surprisingly, identifying a host factor responsible for only a specific step is equally difficult. For instance, a key enigma during DENV infection is the identity of host factors that support the folding and assembly of the virus structural proteins that enable proper genome packaging. In this manuscript, using loss-of-function strategies, our results indicate that the Sigma1 ER membrane receptor (S1R) plays an important role in DENV infection. We found that depletion of S1R impairs formation of intracellular infectious DENV without compromising polyprotein translation or virus replication. Electron microscopy (EM) experiments reveal that the DENV particles in and secreted from S1R-depleted cells are smaller, likely because the defective virions are empty particles devoid of the vRNA genome. Biochemical studies demonstrate that S1R interacts with prM and under S1R-depletion, the prM, E and C structural proteins became detergent-insoluble. Thus, in the absence of S1R, all three virus structural proteins misfold, preventing efficient genome packaging. These findings are consistent with a role of S1R as a chaperone that promotes folding of the virus structural proteins during DENV infection.[10,11]

## Results

### S1R promotes DENV infection

**Fig 1:**
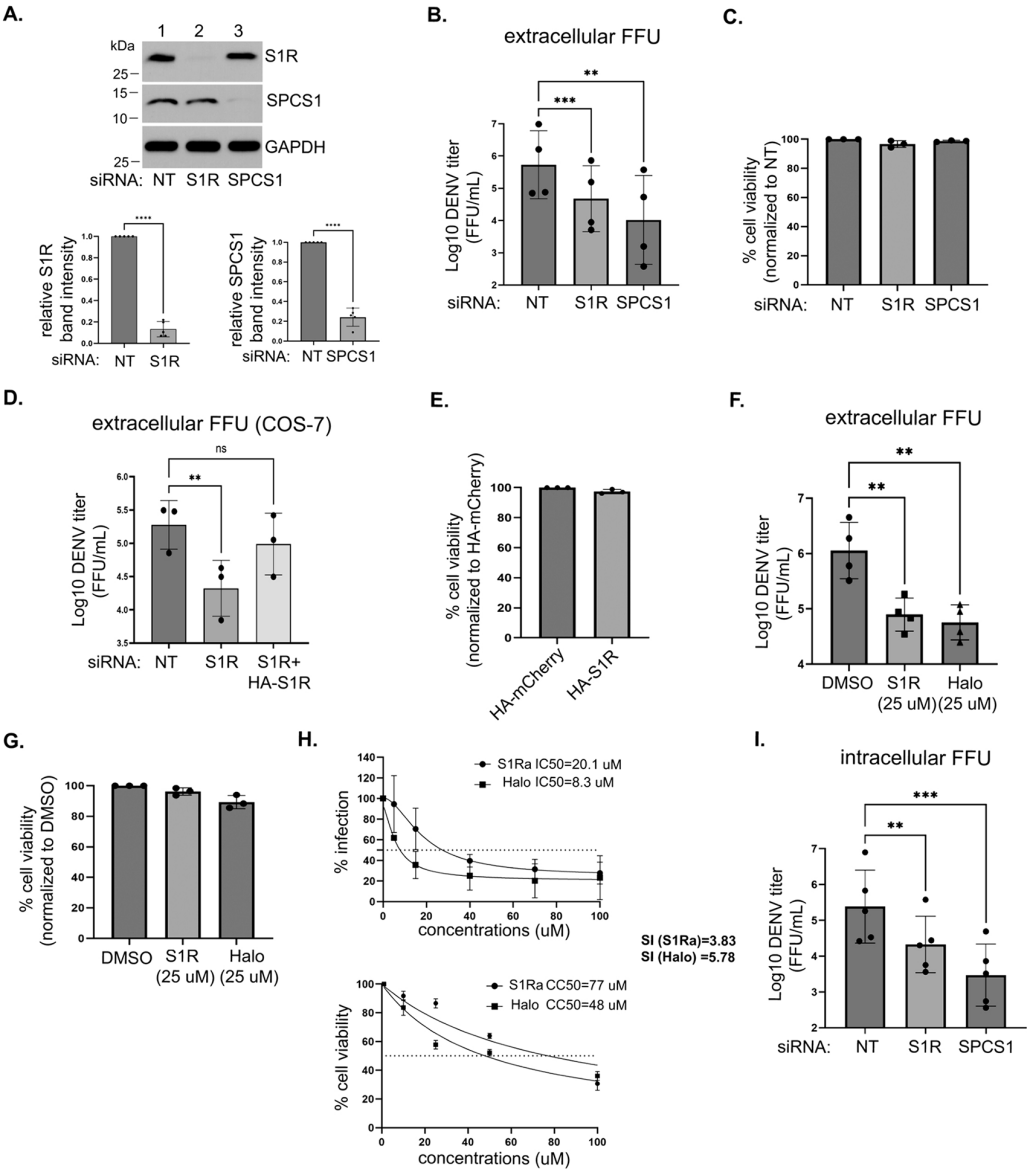
S1R promotes DENV infection. **(A)** Cells were transfected with the control non-targeting (NT), S1R, or SPCS1 siRNA and then infected with DENV (MOI 1). Cells were then harvested 24 hpi and the resulting extracts were subjected to SDS-PAGE and Western blotting. The band intensity of S1R and SPCS1 was quantified by Image J. **(B)** The extracellular virus in the secreted media in A was collected and the infectious titer determined by the focus forming assay. **(C)** To determine cell viability, Huh-7 cells were plated at a density of 2X10^4^ cells per well, transfected with control (i.e., NT) siRNA, or siRNA against S1R or SPCS1, followed by incubation with the MTS reagent for 1 h at 37°C. The absorbance was measured at 490 nm using a multi-mode plate reader. Cell viability was calculated as a percentage of NT-transfected cells. **(D)** Parental COS-7 cells and COS-7 cells expressing HA-S1R were subjected to siRNA treatment (NT/S1R). Post-siRNA transfection, cells were infected with DENV (MOI 1) for 24 h and the supernatant was used to determine titer by focus-forming assay. **(E)** COS-7 cells were transfected with HA-mCherry (control) or HA-S1R. 24 h post-transfection, cell viability was assessed using the MTS assay. Cell viability is expressed as a percentage of cells expressing HA-mCherry. **(F)** Huh-7 cells were pre-treated with the S1R inhibitors S1Ra (25 µM) and Haloperidol (25 µM; Halo) for 12 h. Cells were then infected with DENV (MOI 1) in the presence of the inhibitors and the infectious titer in the secreted media was determined 24 hpi as in A. **(G)** Huh-7 cells treated with the DMSO control or the S1R inhibitors S1Ra or Halo (25 µM) were incubated with the MTS reagent to assess cell viability. Cell viability is expressed as a percentage of DMSO-treated cells. **(H)** For determining the 50% inhibitory concentration (IC_50_), Huh-7 cells were seeded in 24-well plates at a density of 1X10^5^ cells per well and treated with the S1R inhibitors (S1Ra or Halo) at the indicated concentrations (0, 5, 15, 40, 70, and 100 µM) for 12 h. Cells were then infected with DENV (MOI 1) for 48 h in the presence of the inhibitors. IC_50_ was determined based on the FFU assay. DMSO-treated cells served as the vehicle control. For determining the 50% cytotoxic concentration (CC_50_), Huh-7 cells were seeded in 96-well plates at a density of 2X10^4^ cells per well and treated with the S1R inhibitors (S1Ra or Halo) at the indicated concentrations (10, 25, 50, and 100 µM) for 12 h. Cells were then infected with DENV (MOI 1) for 48 h in the presence of the inhibitors. CC_50_ was determined based on the MTS assay. DMSO-treated cells served as the vehicle control. The selectivity index (SI) is defined as CC_50_/IC_50_. **(I)** DENV-infected cells were collected and the intracellular virus isolated as described.[21,22] The intracellular virus was released by using the freeze-thaw method and the resulting clarified supernatant was used for the focus-forming assay. Results are representatives of 3 or more independent experiments. The statistical significance was determined by one-way ANOVA. ***p < 0.0005, **p < 0.005.

Because S1R supports infection by RNA viruses including hepatitis C virus (HCV) [12] and SARS-CoV-2 [13,14], we asked if this ER membrane receptor regulates DENV infection. To test a role of S1R in DENV (i.e., DENV-2 strain) infection, we used an siRNA-mediated knockdown (KD) strategy in Huh-7 cells, a human liver cell line used widely to study DENV infection.[15] When compared to cells transfected with the control non-targeting (NT) siRNA, the level of S1R decreased robustly with siRNA knockdown (Fig 1A, top blot, compare lane 2–1; the S1R band intensity is quantified in graph below); we have previously demonstrated that this S1R siRNA markedly depleted S1R in Huh-7 cells.[16] Because the ER-resident protease SPCS1 plays an important role in DENV replication and infection [17], we also depleted this host factor separately as a positive control (Fig 1A, second blot, compare lane 3–1; the SPCS1 band intensity is quantified in graph below). Infectivity was measured by a focus-forming unit (FFU) assay. Importantly, the level of infectious DENV secreted into the extracellular media of S1R-depleted cells decreased when compared to control cells transfected with the NT siRNA (Fig 1B, compare second to first column); as expected, extracellular infectious DENV also decreased under SPCS1 KD (Fig 1B, compare third to first column). As a control, we performed the MTS cell viability assay to determine cellular integrity and found that overall cellular viability was not perturbed under S1R or SPCS1 KD (Fig 1C).

We next performed a rescue experiment and found that expression of a siRNA-resistant HA-tagged S1R construct (HA-S1R) largely restored the decrease in virus infection under S1R KD (Fig 1D, compare third to second bar). Expression of HA-S1R (or a control protein HA-mCherry) did not affect cell viability (Fig 1E). These findings demonstrate that the decrease in DENV infection resulting from the S1R siRNA is due to depletion of S1R and not to unintended off-target effects. Of note, this rescue experiment was performed in COS-7 cells—known to support DENV infection [18]—because these cells can support high transfection efficiency required for the rescue experiment. In conjunction with the genetic KD experiments, we used the established S1R chemical inhibitors S1Ra [19] and haloperidol (Halo) [20] and found that they blocked DENV infection (Fig 1F) without compromising cell viability (Fig 1G). The 50% inhibitory concentration (IC_50_) values (determined using the FFU assay) and 50% cytotoxic concentration (CC_50_) values (determined using the MTS cell viability assay), as well as the selectivity index (SI) defined as CC_50_/IC_50_, of S1Ra and Halo are shown in Fig 1H. Together, these results demonstrate that S1R promotes DENV infection.

To determine whether the level of intracellular infectious DENV decreased under S1R KD, intracellular virus was isolated from the infected cells by freeze-thaw [21,22] and titered by FFU measurement. Again, depletion of S1R decreased the level of intracellular infectious DENV in S1R-depleted cells when compared to control (Fig 1I, compare second to first column), similar to the decreased level of extracellular infectious DENV observed under S1R KD (Fig 1B); KD of SPCS1 also decreased intracellular infectious DENV (Fig 1I, compare third to first column), as anticipated.[17] This data strongly suggest that S1R promotes formation of infectious DENV in the cell.

### DENV polyprotein translation or replication does not depend on S1R

**Fig 2:**
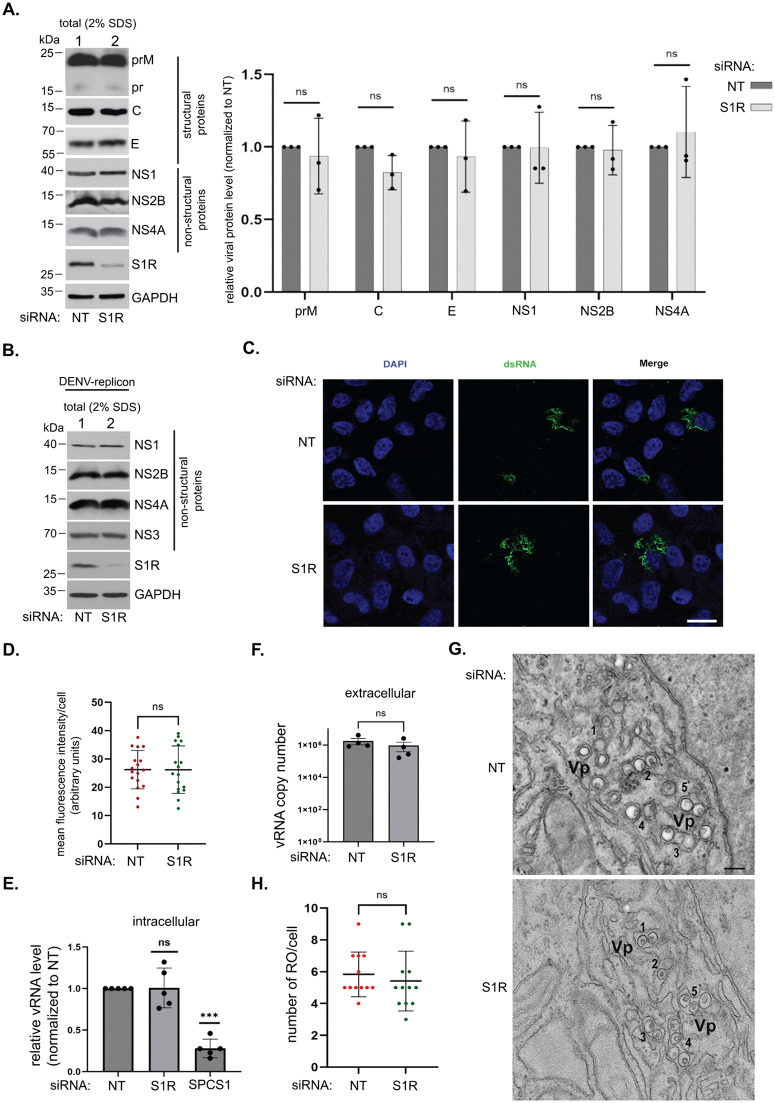
DENV polyprotein translation or replication does not depend on S1R. **(A)** The levels of the viral structural and non-structural proteins remain unaffected by S1R depletion. Huh-7 cells transfected with the indicated siRNA were infected by DENV (MOI 1). Total proteins from the infected cells were extracted 24 hpi using 2% SDS and the extracts analyzed by SDS-PAGE and Western blotting. Right graph: the band intensity of the virus proteins was quantified by Image J. **(B)** Huh-7 cells harboring a subgenomic DENV replicon was transfected with the indicated siRNA and the resulting cell lysates were subjected to Western blotting 2 days later using the indicated antibodies. **(C)** Control and S1R-depleted DENV-infected (MOI 1) cells were stained by DAPI (blue) at 24 hpi to mark the nucleus and a dsRNA antibody (green) to mark active replication. Scale bar: 10 µm **(D)** The mean fluorescence intensity per cell in C was quantified. **(E)** The levels of the total intracellular vRNA in control and S1R-depleted cells were quantified by qRT-PCR and the levels were normalized to the control cells. **(F)** To determine extracellular vRNA, Huh-7 cells were treated with siRNA (NT/S1R). Cells were then infected with DENV (MOI 1) for 24h and the harvested supernatant subjected to RNA isolation. Viral copy number in the supernatant was determined against the DENV capsid standard curve using RT-qPCR. **(G)** TEM analysis was performed on DENV-infected control and S1R KD cells. The images showed a comparable number of RO containing Vps in control and S1R KD cells. A RO is defined as containing 2 or more individual Vps. **(H)** Quantification of the number of replication organelles (RO) per cell in 12 independent randomly selected cells in G. Statistical significance was determined by students t-test. ns, not significant. Scale bar: 200 nm.

Because S1R is localized at the ER where translation of the viral polyprotein occurs [9,10,23,24], we asked whether S1R affects virus translation by assessing the levels of virus structural and non-structural proteins in DENV-infected control and S1R-depleted cells. The virus-infected cells were harvested and treated with 2% SDS to extract all proteins in the cell (i.e., total) and the samples subjected to SDS-PAGE followed by Western blotting. Under S1R KD (quantified in Fig 1A), the levels of the viral structural proteins prM (and its cleaved product pr), C and E, as well as the nonstructural NS1, NS2B and NS4A, did not decrease when compared to control cells (Fig 2A; the levels of the proteins are quantified in the right graph), suggesting that DENV polyprotein translation was unaffected by S1R depletion. To further support this finding, we found that expression of NS1, NS2B, NS3, and NS4B in Huh7.5.1 cells stably expressing a DENV replicon [24], which expresses virus NS but not structural proteins, was similarly unaffected under S1R KD (Fig 2B, compare lane 2–1). These results indicate that translation of DENV NS proteins does not depend on S1R.

We then tested whether replication of DENV depends on S1R. DENV-infected cells were stained with an antibody against double-stranded (ds) RNA, which marks active DENV replication.[25] We found that depletion of S1R did not impair appearance of the DENV dsRNA signal when compared to control cells (Fig 2C, compare bottom to top row; the mean fluorescence intensity/cell is quantified in Fig 2D), indicating that DENV replication remains intact under S1R KD. In addition, we used quantitative RT-PCR (qRT-PCR) to measure intracellular vRNA and found that the vRNA level did not decrease under S1R KD (Fig 2E), consistent with dsRNA staining (Fig 2C and 2D); in contrast, the vRNA under SPCS1 KD decreased because this host factor supports DENV replication [17]. Additionally, under S1R KD, the level of extracellular vRNA was unaffected when compared to control cells (Fig 2F), suggesting that secretion of vRNA is not disrupted by loss of S1R. Finally, we performed transmission electron microscopy (TEM) experiments to directly examine if DENV-induced formation of vesicle packets (Vp; an ER membrane invagination believed to be the site of DENV genome replication) is perturbed by depleting S1R and found that it was not (Fig 2G; the number of replication organelle RO/cell is quantified in Fig 2H). Taken together, these findings indicate that S1R supports a post-replication step during DENV infection.

### The assembled DENV particles are smaller in S1R-depleted cells

**Fig 3:**
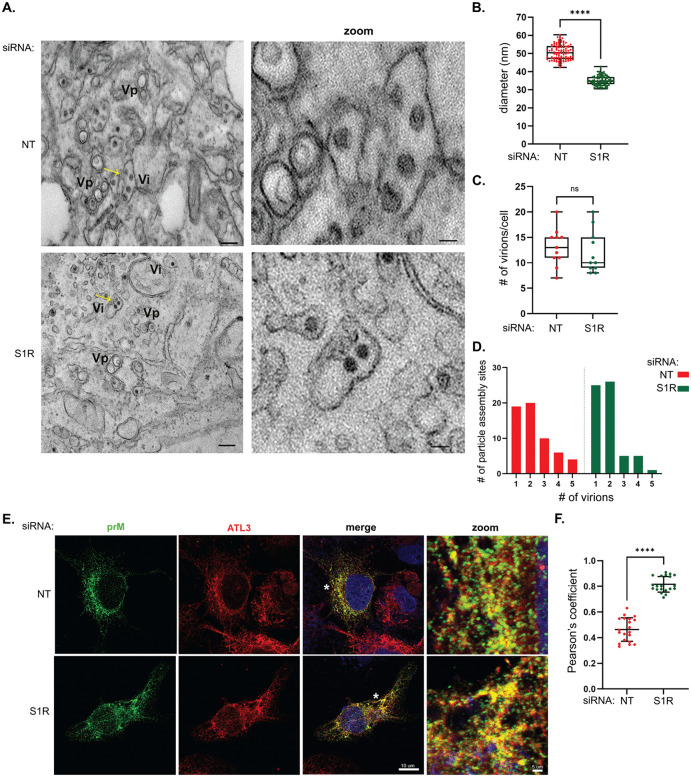
The assembled DENV particles are smaller in size in S1R-depleted cells. **(A)** TEM analysis was performed on DENV-infected control and S1R KD cells. Vesicle packet (Vp), DENV Virions (Vi). Yellow arrow indicates the magnified region. Scale bar: 200 nm. **(B)** Quantification of the diameters of the DENV particles observed in A. Each dot represents a single virion. NT = 50.7±4 nm, and S1R KD = 35.0±3 nm. (n = 100) **(C**) EM images were quantified to determine the number of virions per cell under siRNA-treated (NT or S1R) conditions. Each data point represents an individual cell analyzed per condition (n = 11). **(D)** EM images were analyzed to quantify the number of particle assembly sites (i.e., dilated ER cisternae) and the number of virions per assembly site under NT or S1R condition. The data represent the quantitative measurements from the analyzed EM micrographs under each condition. **(E)** Confocal imaging of the DENV structural protein prM (green) and the ER-assembly site marker ATL3 (red) in control and S1R-depleted cells infected with DENV (MOI 1) for 24h. Scale bar: 10 µm **(F)** Quantification of colocalization using images in C by Pearsons’ colocalization coefficient. n = 20. Each dot represents a single field. Statistical significance was determined by students t-test. ****p < 0.0001.

We used TEM to visualize the assembly state of DENV in control and S1R KD cells. In virus-infected control cells, the newly-assembled particles are approximately 50.7 ± 4 nm in diameter (Fig 3A, top, see yellow arrow and the corresponding zoom image; the diameter of the DENV is quantified in 3B), as previously reported.[6,7] Additional images of DENV in control cells, along with measurements of the virus diameter, are shown in S1A Fig Strikingly, in DENV-infected S1R-depleted cells, the diameter of the newly assembled virus was noticeably smaller, with a mean diameter of 35.0 ± 3 nm (Fig 3A, bottom, see yellow arrow and the corresponding zoom image; the diameter of the DENV is quantified in 3B), again with additional DENV images and virus diameter measurements shown in S1B Fig. Not surprisingly, Vps are observed in both DENV-infected control and S1R KD cells (Fig 3A, top and bottom, Vp), consistent with Fig 2G and 2H. Although the virions in S1R KD cells are smaller, the total number of virions per cell was unaffected by S1R-depletion (Fig 3C). We further characterized the abundance of virions in dilated ER cisternae that are generally thought to represent particle assembly sites, as well as the number of virions per assembly site, and found only small differences between control and S1R KD cells (Fig 3D). For instance, in control cells, there were approximately 20 ER sites containing 1 or 2 virions, while in S1R KD cells, there were approximately 23 ER sites harboring 1 or 2 viral particles. Nonetheless, the key observation that smaller DENV particles are found in S1R-depleted cells suggests that S1R regulates the size of the DENV particles, which likely accounts for the reduced infectivity observed in the S1R KD cells (Fig 1I).

The ER morphogenic protein Atlastin3 (ATL3) was previously shown to play a role in DENV infection, possibly by promoting formation of a virus assembly site.[21] By confocal microscopy, we found that the structural protein prM colocalizes with ATL3 (Fig 3E, top; the extent of prM-ATL3 colocalization is quantified in 3F). Under S1R KD, the level of prM-ATL3 colocalization increased (Fig 3E, bottom; quantified in 3F), suggesting that prM accumulates in the ER-associated assembly site under S1R KD. If this model is true, we would expect impaired trafficking of prM to the Golgi. Indeed, co-localization of prM with the TGN marker TGN46 decreased under S1R KD (S1C Fig; quantified in S1D Fig).

### S1R binds to prM and promotes solubility of the DENV structural proteins

**Fig 4:**
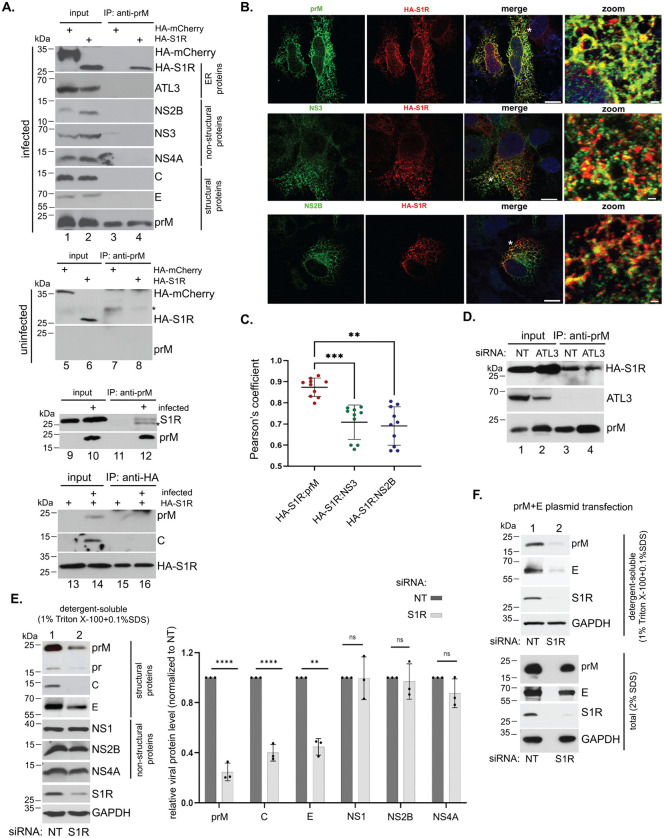
S1R binds to prM and promotes solubility of the DENV structural proteins. **(A) (lanes 1–4)** HEK293T cells were transfected with the control HA-mCherry or HA-S1R plasmid 24 h prior to infection. Cells were infected with DENV (MOI 10) for 24 h, lysed, and the resulting extract subjected to co-immunoprecipitation using an anti-prM 6.1 antibody. The precipitated material was subjected to Western blotting using the indicated antibodies. Presence of HA-S1R in lane 4 indicates interaction with prM. **(lanes 5–8)** HEK293T cells transfected with HA-mCherry (control) or HA-S1R plasmids were lysed, and the resulting extracts subjected to co-immunoprecipitation using the anti-prM 6.1 antibody. The immunoprecipitated material was analyzed by SDS-PAGE and Western blotting with the indicated antibodies to assess the specificity of the prM–S1R interaction. The asterisk (*) denotes a non-specific band. **(lanes 9–12)** Mock-or DENV-infected (MOI 1, 24h) Huh-7 cells were lysed and the resulting extract subjected to co-immunoprecipitation using the anti-prM 6.1 antibody. The immunoprecipitated material was subjected to SDS-PAGE and Western blotting with the indicated antibodies to determine interaction between prM and endogenous S1R. The asterisk (*) denotes a non-specific band. **(lanes 13–16)** HEK293T cells transfected with HA-S1R was mock or DENV-infected (MOI 10, 24h). Cells were lysed and the resulting extract subjected to co-immunoprecipitation using an anti-HA antibody. The immunoprecipitated samples were subjected to SDS-PAGE and Western blotting with the indicated antibody. **(B)** Huh-7 cells expressing HA-S1R were infected with DENV (MOI 1, 24 h), stained with HA (red), and either prM (green), NS3 (green), or NS2B (green), and analyzed by confocal microscopy. Scale bar: 10 µm. **(C)** Quantification of the extent of colocalization of HA-S1R:prM, HA-S1R:NS3, and HA-S1R:NS2B using Pearson’s coefficient. **(D)** HEK 293T cells were transfected with NT or ATL3 siRNA for 24 h, followed by transfection of the HA-S1R plasmid. 24 h post-plasmid transfection, the cells were infected with DENV (MOI 10) for 24 h, lysed, and the resulting extract was subjected to co-immunoprecipitation using the anti-prM 6.1 antibody. The precipitated material was subjected to SDS-PAGE and western blotting using the indicated antibodies. **(E)** Control and S1R-depleted cells were infected with DENV and the detergent-soluble proteins from the cells extracted using RIPA buffer containing 1% Triton X-100 and 0.1% SDS. The extracted materials were subjected to Western blot analysis using the indicated antibodies. Right graph: the band intensity of the virus proteins was quantified by Image J. **(F)** Huh-7 cells transfected with the NT or S1R siRNA were additionally transfected with a plasmid encoding prM and E proteins. Cells were lysed using either RIPA buffer (containing 1% Triton X-100 and 0.1% SDS) or 2% SDS. The resulting extracts were subsequently analyzed by SDS-PAGE and Western blotting with the indicated antibodies. Statistical significance was determined by students t-test. ****p < 0.0001, ***p < 0.0005, **p < 0.005.

Because S1R has been proposed as a molecular chaperone [26], we hypothesize that this host factor deploys its chaperone activity, interacting with the DENV structural proteins to support their folding and/or assembly. We therefore tested whether S1R physically interacts with the DENV structural proteins. HEK293T cells (which have a high transfection efficiency) were transfected with HA-tagged mCherry (control) or HA-tagged S1R (HA-S1R). The cells were then infected with DENV and the resulting cell extract subjected to immunoprecipitation (IP) using an anti-prM 6.1 antibody. We found that IP of prM pulled down HA-S1R, but not (as negative controls) HA-mCherry, ATL3, or the viral proteins NS2B, NS3, NS4A, C and E (Fig 4A, compare lane 4–3 in the top blot to rest of blots), demonstrating that S1R binds to prM. The anti-prM antibody did not pulldown HA-S1R if an uninfected cell extract was used (Fig 4A, compare lane 8–7 in top blot), indicating that presence of prM in the infected cells is required for the anti-prM antibody to precipitate HA-S1R. Importantly, the anti-prM antibody precipitated endogenous S1R from an infected (but not uninfected) cell extract (Fig 4A, compare lane 12–11 in top blot), demonstrating that the prM-S1R interaction is unlikely an overexpression artifact. We then performed a reverse coIP experiment using extracts from infected cells and found that IP of HA-S1R did not pull down prM (or C) (Fig 4A, compare lanes 15 and 16 in first and second blots). This suggests that the majority of S1R did not interact with prM, but instead only a small pool of S1R binds to the prM structural protein during infection.

Confocal microscopy revealed that prM strongly colocalizes with HA-S1R, while the NS3 and NS2B ER-associated nonstructural proteins (localized to the replication complex) show lower co-localization with HA-S1R (Fig 4B, compare zoom in top row to second and third rows; quantified in 4C). These findings further support the idea that prM binds to S1R. The S1R-prM interaction is retained under ATL3 KD (Fig 4D, compare lane 4–3 in top blot), indicating that this interaction occurs independent of ATL3.

By binding to a substrate, a chaperone helps to maintain solubility of the substrate, thereby preventing the substrate from forming detergent-insoluble aggregates. Because S1R interacts with prM (Fig 4A), we asked whether S1R enables this DENV structural protein to remain soluble. To test this, we used a mix of detergents (i.e., 1% Triton X-100 + 0.1% SDS)—which readily extracts soluble proteins in cells—to extract virus proteins from DENV-infected control and S1R KD cells. We find that prM, as well as the C and E, structural proteins cannot be readily extracted by this detergent in S1R-depleted cells when compared to control cells (Fig 4E, top three blots; quantified in right graph), even though the total amounts of these structural proteins (extracted by using the harsh detergent 2% SDS) are the same between control and S1R KD cells (Fig 2A). In contrast, the detergent extractability of NS1, NS2B, and NS4A is unaffected by S1R KD (Fig 4E, 4^th^–6^th^ blots; quantified in right graph). These findings indicate that the solubility of DENV structural proteins (but not nonstructural proteins) depends on S1R, strongly suggesting that S1R promotes folding of the virus structural proteins. We further evaluated the detergent solubility of transfected prM and E proteins using a plasmid encoding the prM and E proteins. Again, while there is only a modest decrease in expression of prM and E proteins in S1R-depleted cells (Fig 4F, 5^th^ and 6^th^ blots, compare lane 2–1), both of these structural proteins cannot be efficiently extracted by detergent under S1R KD (Fig 4F, compare lane 2–1 in first and second blots). Hence, S1R promotes detergent solubility of the DENV structural proteins not only during infection, but also during transfection of the virus proteins.

### Characterization of the defective secreted DENV particles from S1R-depleted cells

**Fig 5:**
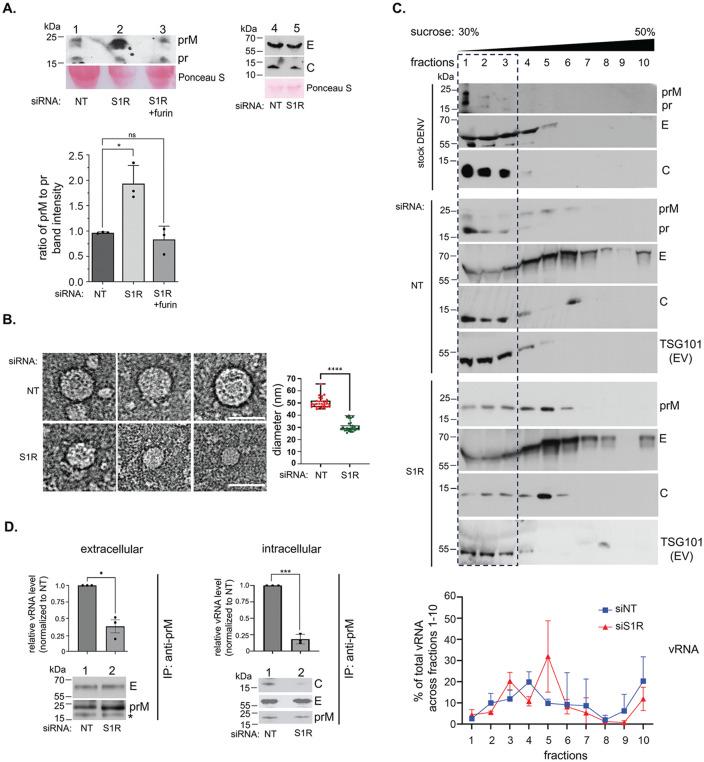
Characterization of defective secreted DENV particles from S1R-depleted cells. **(A) (lanes 1-2)** The media harboring the secreted virus from control or S1R-depleted cells (infected with DENV) was clarified, concentrated, and subjected to SDS-PAGE followed by either Western blotting with the anti-prM antibody or Ponceau S staining. **(lane 3)** The concentrated media from S1R-depleted cells was incubated with furin and the sample subjected to SDS-PAGE and Western blotting using the anti-prM antibody. **(lanes 4–5)** The concentrated media from control or S1R-depleted cells (infected with DENV) was subjected to SDS-PAGE followed by Western blotting with Envelope or Capsid antibody, or Ponceau S staining. The bottom graph represents the ratio of prM to pr band intensities in lanes 1–3. **(B)** The media from control and S1R-depleted cells (infected with DENV) was clarified, concentrated, and subjected to negative-stain EM to visualize the secreted virus. Three representative examples under each condition are shown. The graph represents the quantification of the diameter (nm) of the secreted virus. NT = 50.2±4 nm, and S1R KD = 30.4±4 nm. (n = 31) Scale bar: 50 nm **(C)** Concentrated media from control or S1R-depleted cells (infected with DENV) was layered on a 30–50% continuous sucrose gradient, centrifuged, and the individual fractions collected and analyzed by SDS-PAGE and Western blotting using the indicated antibodies. A stock virus was subjected to the same sucrose gradient fractionation protocol. The vRNA level across all the sucrose fractions was analyzed by RT-qPCR. The dotted line box indicates fractions containing the assembled virus. **(D)** (left: extracellular) Concentrated media from control or S1R-depleted Huh-7 cells (infected with DENV) was subjected to co-immunoprecipitation using the anti-prM 6.1 antibody. The resulting precipitated materials were analyzed by SDS-PAGE and Western blotting and RNA isolation to determine the level of prM-associated viral protein and viral RNA. The asterisk (*) denotes a non-specific band. (right: intracellular) Intracellular virus isolated from control and S1R KD cells by the freeze-thaw method was subjected to the same analysis as the extracellular virus. Statistical significance was determined by students t-test. ****p < 0.0001, ***p < 0.0005, **p < 0.005, *p < 0.05.

Because the DENV structural proteins became detergent-insoluble under S1R KD (Fig 4E), we tested whether these misfolded virus proteins are secreted. In the media of control cells, we found prM and its cleaved product pr (Fig 5A, lane 1 in top blot); the ratio of the prM to pr band intensities suggests they are at a similar level (see quantification in the bottom graph). By contrast, more prM than pr was observed in the media of S1R-depleted cells when compared to control cells (Fig 5A, lane 2 in top blot; the ratio of the prM to pr band intensities is quantified in the bottom graph), indicating prM was inefficiently cleaved in the infected (S1R KD) cells prior to secretion. This result is consistent with the decreased co-localization of prM with the TGN (where furin-mediated cleavage occurs) under S1R KD (S1C and S1D Fig). When exogenous furin was incubated with media from S1R-depleted cells, the ratio of prM to pr is similar to control cells (Fig 5A, lane 3 in top blot and quantifed in the bottom graph), demonstrating that extracellular prM is competent for furin cleavage. The E protein was efficiently secreted under S1R KD when compared to control cells (Fig 5A, compare lane 5–4 in top blot), while secretion of the C protein modestly decreased (Fig 5A, compare lane 5–4 in second blot). Thus, despite the DENV structural proteins all becoming misfolded under S1R KD (Fig 4E), secretion of prM and E remains unimpeded with C protein secretion being moderately affected.

We next determined whether DENV particles are assembled when they are secreted by S1R-depleted cells. Again, by TEM, we found that the DENV particles secreted from S1R-depleted cells can be assembled but are smaller when compared to control cells (Fig 5B, compare examples in bottom to top row), with virus released from S1R-depleted cells measuring approximately 30 ± 4 nm in diameter while those from control cells are 50 ± 4 nm in diameter (Fig 5B, right graph). These measurements are similar to those observed within the infected cells (Fig 3A and 3B).

Sucrose gradient analysis was used to further characterize the gross structural state of the virions secreted from control and S1R-depleted cells; we used stock DENV as a positive control because this virus should be assembled. The majority of the stock DENV—as assessed by the fractionation pattern of prM/pr, E, and C proteins (Fig 5C, top three blots, dotted line box)—were detected in fractions 1–3, indicating that these fractions harbor assembled virions. Importantly, the majority of the C and cleaved prM (i.e., pr) proteins, as well as some of the E protein, secreted from control cells were found in fractions 1–3 (Fig 5C, blots 4–6). Thus, DENV particles in these fractions represent assembled virus.

By contrast, the fractionation pattern of the C and prM structural proteins secreted from S1R KD cells was different. Specifically, low level of the C protein distributed across fractions 1–6, with accumulation in fraction 5 (Fig 5C, 10^th^ blot), and a low level of the prM protein also distributed across fractions 1–6 (Fig 5C, 8^th^ blot); no difference was observed in the fractionation pattern of the E protein (Fig 5C, compare 9^th^ to 5^th^ blot). The broad distribution of the C and prM proteins across more fractions indicates that virions secreted from S1R KD cells exist in a conformation distinct from the control virus, consistent with the EM findings (Fig 5B). Distribution of the C and prM proteins to denser sucrose fractions (i.e., fractions 4–6) under S1R KD may be explained by their propensity to aggregate because they are detergent-insoluble. We then measured the vRNA level across the sucrose fractions by RT-qPCR (Fig 5C, bottom graph). While a detectable level of vRNA is found in all fractions in control and S1R KD cells, there appears to be an elevated vRNA level in fraction 5 under S1R KD, where the C and prM protein levels are also the highest. Thus, a pool of vRNA likely accumulates with the aggregated virus structural proteins.

Finally, to explain the decreased in size of the secreted virus under S1R KD, we performed coIP experiments. Strikingly, we found that IP of prM pulled down less vRNA in the extracellular (i.e., secreted) virus from S1R-depleted cells when compared to control cells (Fig 5D, left, top graph), whereas the level of E protein that co-precipitated with prM was unaffected (Fig 5D, left, top blot, compare lane 2–1); we did not detect the C protein in the precipitated material even in control cells. Consistent with this finding, IP of prM also pulled down less vRNA using intracellular virus from S1R-depleted cells when compared to control cells (Fig 5D, right, top graph); in this case, the C protein did co-precipitate with prM in the control cells. A lower level of the C protein was pulled down in S1R KD cells when compared to control cells (Fig 5D, right, top blot, compare lane 2–1); this is anticipated given the level of vRNA—which associates directly with the C protein during assembly—that precipitates with prM also decreased under S1R KD. The level of E protein co-precipitating with prM was unaffected by S1R KD (Fig 5D, right, second blot, compare lane 2–1). Because DENV particles devoid of the RNA genome are reported to be smaller and approximately 30–35 nm in diameter [27–29], one likely explanation for the decreased size of virions secreted from S1R-depleted cells is that the defective virus is an empty particle lacking its vRNA genome. Thus, S1R-dependent folding of the virus structural proteins enables the efficient packaging of the vRNA.

We note that the level of extracellular vRNA secreted from S1R-depleted cells did not decrease (Fig 2F). This could be because the great majority of the extracellular vRNA are not packaged with the virus and are instead secreted separately from the virus. This possibility is consistent with presence of extracellular vesicles (EVs)—as indicated by the marker TSG101 in the secreted media of both control and S1R KD cells (Fig 5C, 7^th^ and 11^th^ blots) —which have been reported to mediate secretion of unpackaged DENV vRNA [30]. Hence, in addition to the pool of vRNA that accumulates with the aggregated prM/capsid proteins (i.e., fraction 5), the vRNA pool co-fractionating with TSG101 (i.e., fractions 1–3) may be secreted by extracellular vesicles.

## Discussion

Because virus protein translation, folding, replication, assembly, and genome packaging of DENV all rely on the host ER to varying extent, elucidating and uncoupling the mechanism of these different infection steps, as well as identifying the specific ER components that are exploited to execute these events, are challenging. In this manuscript, we identify a host factor that plays a selective role in supporting virus structural protein folding and genome packaging of DENV.

Using genetic and chemical loss-of-function strategies coupled to a rescue approach, we found that S1R plays an important role in DENV infection. Specifically, the levels of both intracellular and secreted infectious DENV decreased when S1R activity is disrupted, suggesting that S1R is important for generating infectious intracellular virus. Under S1R KD, virus protein translation and replication of DENV were not affected. Instead, TEM experiments revealed that DENV particles in and secreted from S1R-depleted cells are assembled but appear smaller (ranging from 30-35 nm in diameter), in contrast to the 50 nm virus particles found in and secreted from control cells, which is the native size of the virus.[6,7] Importantly, binding studies (using both intracellular and extracellular virus) suggest that the smaller size of the DENV particles is likely due to the particles missing their vRNA genome. Additional biochemical studies showed that S1R binds to prM, and in S1R-depleted cells, prM, E, and C became detergent-insoluble indicative of protein misfolding. Collectively, these data suggest that S1R supports folding of DENV structural proteins to enable efficient packaging of the vRNA genome.

How might S1R-dependent folding of the DENV structural proteins play a role in genome packaging? Mechanistically, S1R has been proposed to have numerous biological activities, including a chaperone function.[10,11] As a chaperone, S1R could assist folding of prM and E so that natively-folded prM and E can reorganize on the ER membrane and oligomerize. This oligomerization step induces ER membrane invagination which leads to budding of the viral particles into the ER lumen [7]. Because the C protein-vRNA genome complex is thought to be passively engulfed during the budding process, it is possible that misfolding of prM and E indirectly impairs either prM-E oligomerization, membrane curvature induction, or budding which preclude vRNA packaging. Because the DENV particle still assembles under S1R KD, any misfolding of prM and E in S1R-depleted cells must still allow these structural proteins to interact, as we observed (Fig 5D). Intriguingly, S1R was shown to possess RNA-binding activity [16], suggesting that it may actively package the vRNA into the DENV particle during virus budding. In this scenario, loss of S1R would directly disrupt genome packaging, leading to formation of the smaller DENV particles.

In addition to acting as a chaperone and an RNA-binding protein, S1R also promotes formation of cholesterol-rich microdomains in the ER [31]. In this context, the prM of the flavivirus Zika virus was recently shown to have a cholesterol-binding motif that mediates assembly [32]. These observations leave open the possibility that S1R-dependent regulation of cholesterol-rich ER subdomains might impact prM assembly.

Although we found the S1R binds to prM during infection, whether the S1R-prM interaction is direct remains unclear. Because the ER-resident chaperone BiP, a Hsp70 family member, was previously shown to be a S1R binding-partner [26], it is possible that BiP participates in the S1R-prM interaction. BiP and the ER luminal chaperones calnexin and calreticulin were previously reported to participate in DENV assembly by associating with the E protein.[33] Interestingly, the ER luminal ERdj3/DNAJB11 co-chaperone, a J-protein that stimulates the BiP ATPase activity, plays a role in DENV infection, except in this case ERdj3 assists in virus replication instead.[34]

There are limited number of reports that have identified ER factors that are dedicated to promoting DENV protein translation, folding, replication, assembly, genome packaging, as well as ER exit. For instance, the Sec61-EMC complex was shown to be important for translation of the DENV vRNA [23,24], the GTPase ATL2 in virus replication [21], the GTPase ATL3 in virus assembly [21], and the KDEL receptor in ER exit of the virus [22]. Our study here identifies S1R in virus structural protein folding and genome packaging of DENV.

Finally, DENV remains a significant global health concern, especially because of the lack of anti-viral therapeutics against DENV infection. Our study here establishes a critical host component in DENV infection, potentially opening new opportunities to rationally develop anti-DENV drugs. For instance, because we found that the FDA-approved S1R inhibitor S1Ra markedly blocked DENV infection, S1Ra may serve as an appropriate starting compound that leads to the development of effective anti-DENV therapeutics.

## Materials and methods

### Cell lines and virus production

The human hepatoma carcinoma Huh-7 cell line was purchased from the Japanese Cancer Research Resources Bank (JCRB), and the Vero, COS7, and HEK 293T cells were purchased from ATCC. Cells were maintained in high-glucose Dulbecco’s modified Eagle’s medium (DMEM) supplemented with 10% fetal bovine serum (FBS) and 2% penicillin/streptomycin (PS). The mosquito cell line C6/36 was grown in DMEM supplemented with 10% FBS and 2% PS at 28°C. To generate DENV (16681 strain, DENV serotype 2, DENV-2), C6/36 cells were infected at 0.2 MOI for 72 h at 28°C, before harvesting the supernatant and the collected virus was stored at -80°C.

### Antibodies, reagents and siRNA

The following antibodies, DENV NS2B (GTX124246), NS1 (GTX103346), NS4A (GTX132069), NS3 (GTX124252), Envelope (GTX127277) and prM (GTX128093), were purchased from Genetex. The dsRNA antibody (J2) was purchased from Jena Biosciences. DENV anti-prM 6.1 and Capsid (clones 2G11 and D2-C2) antibodies were kind gifts from Dr. Chunya Puttikhunt. DENV prM hybridoma cell line (prM12, prM13 and prM 22) was a generous gift from Dr. Michael Diamond. Custom-designed siRNA for S1R, anti-HA antibody (H6908), Amicon Ultra Centrifugal Filter (UFC5100), and HRP-conjugated secondary antibodies were obtained from Sigma Aldrich. Inhibitors used in the study haloperidol (12014) and S1Ra (16279) were obtained from Cayman Chemicals. Fluorescent-labelled secondary antibodies (anti-mouse A-11029, anti-rabbit A-11037), ProLong Gold with DAPI (P36935), and transfection reagents Lipofectamine 2000 (11668019), RNAiMAX (13778150) and Pierce Protein A/G Agarose (20421) were purchased from Invitrogen (Thermo Fisher Scientific).

### Virus titration experiments

For the focus-forming assay, Vero cells were infected with 10-fold serial dilutions of infectious supernatants from NT, S1R, and SPCS1 KD samples. The cells were maintained in DMEM supplemented with 10% FBS. At 3 days post-infection, the media was removed, and the cells were fixed with a layer of 2% paraformaldehyde. Post-fixation, the samples were permeabilized with 0.3% Tween-20 in 1% BSA for 15 min, followed by blocking in 1% BSA at room temperature for 1 h. The cells were then stained with primary antibody 4G2 staining for pan-flaviviral envelope. After staining, samples were washed with 1% BSA and incubated for 1 h at room temperature with HRP-conjugated anti-mouse secondary antibody. After washing, the plate was incubated with TrueBlue Peroxidase Substrate (95059–168) solution and stained for 15 min. The viral focus was counted under a light microscope.

### MTS assay

We used the MTS assay (MTS Assay Kit, Abcam, ab197010) to determine cell viability. Huh-7 cells were plated at a density of 2x10^4^ cells per well in a 96-well plate and transfected with siRNA (NT/S1R/SPCS1) or treated with the control DMSO or S1R inhibitors (S1Ra/Halo) for 48 h. Following treatment, cells were incubated with the MTS reagent according to the manufacturer’s protocol at 37°C for 1 h. The plate was briefly shaken, and absorbance (OD = 490 nm) was read using a SpectraMax M5e multimode microplate reader (Molecular Devices). Cell viability was determined relative to the controls. To determine the inhibitory concentration 50 (IC_50_), cells were pretreated with the S1R inhibitors at the indicated concentrations (10, 25, 50, and 100 µM) for 12 h. Cells were infected with DENV (MOI 1) for 48 h in the presence of the inhibitors. DENV-mediated cytopathic effect (CPE) was determined by MTS assay, as described above.

### SDS-PAGE and Western blotting

Huh-7 and DENV-replicon cells [24] were transfected with siRNA prior to harvesting for protein analysis. For detergent-soluble protein extraction, cells were washed with PBS and lysed in RIPA buffer (50 mM Tris pH 7.5, 150 mM NaCl, 1% Triton X-100, 0.1% SDS and 0.5% sodium deoxycholate) supplemented with PMSF on ice for 1 h. Extracts were centrifuged at 16,000 rpm for 5 min, and the supernatant was mixed with 5X sample buffer and boiled for 15 min at 60 °C. Proteins were separated by SDS-PAGE and transferred to PVDF membranes. Membranes were blocked with 3% milk in TBST and incubated overnight at 4 °C with primary antibodies, followed by HRP-conjugated secondary antibodies for 1 h at room temperature. After thorough washing with TBST, blots were developed using ECL substrate, and the signals were detected on X-ray films. For 2% SDS protein extraction, the cells were washed with PBS and incubated with 2% SDS sample buffer for 30 min at 60 °C. Media (i.e., extracellular sample) from NT or S1R-depleted infected samples was concentrated using Amicon Ultra filters (~100 kDa cutoff), and the resulting concentrated sample was incubated with 1X sample buffer, heated at 95 °C for 15 min, and processed as above. Huh-7 cells were transfected with a DENV-2 prME-expressing plasmid (addgene #207264) under non-targeting (NT) or S1R siRNA conditions. At 24 h post-transfection, cells were harvested with either RIPA buffer to extract detergent-soluble proteins or 2% SDS to extract detergent-insoluble proteins, as described above. Protein samples were analyzed by SDS-PAGE and Western blotting using the indicated antibodies.

### Co-immunoprecipitation

For co-IP experiments, HEK293T cells were transfected with the HA-mCherry or HA-S1R plasmid. At 24 h post transfection, the cells were infected with DENV (MOI 10) for 24 h and the cells were lysed using NP-40 lysis buffer (50 mM Tris pH 7.5, 150 mM NaCl, 1mM EDTA,1% NP-40, 0.25% sodium deoxycholate and PMSF). The resulting supernatant was incubated with anti-prM 6.1 antibody overnight at 4 °C. The immune complex was incubated with protein A/G agarose beads for 2 h at room temperature. Following the incubations, the beads were washed with the lysis buffer, and the protein complex was eluted with 1X sample buffer for 30 min at 60 °C. The samples were analyzed by SDS-PAGE and Western blotting. For control and ATL3 depleted co-IP, HEK 293T cells were treated with siRNA followed by transfection of the HA-S1R plasmid. At 24 post-transfection, the cells were infected with DENV (MOI 10), and the resulting samples were subjected to co-immunoprecipitation as above. The extracellular media containing the secreted virus was subjected to co-IP analysis. After concentrating the secreted media from control or siRNA-treated cells (NT/S1R), the samples were incubated with an anti-prM 6.1 antibody as described above and the precipitated material was subjected to SDS-PAGE followed by Western blotting. For IP of the intracellular virus, cells were subjected to freeze thaw and incubated with anti-prM 6.1 antibody as described above.

### RNA extraction and RT-qPCR

RNA from DENV infected cells or the supernatant were isolated using RNeasy kit (Qigen #74104). iScript cDNA synthesis kit (Biorad #1708890) was used to generate the cDNA. Real-Time PCR was set up using SYBR green qPCR supermix (BioRad #1725271). Relative viral RNA level was compared to NT control. GAPDH was used as a housekeeping gene. DENV capsid primers were used to generate a standard curve for viral copy number. (Capsid Forward-5’TAATACGACTCACTATAGGGACGCGAGAGAAACCGCGTGTCGACTGT3’ and Capsid Reverse-5’AAACGAAGGAACGCCACCAGGGCCAGCTAGTTATTGCTCAGCGG3’).

To determine the level of vRNA that binds to the secreted virus, concentrated extracellular media were incubated with the anti-prM 6.1 antibody and the bound material pulled down using protein A/G-agarose beads. The beads were washed and RNA from the samples was isolated using TRIzol reagent (15596026). The RNA was subjected to cDNA synthesis as described above, followed by real-time PCR using the capsid C protein primer.

### Immunofluorescence staining and confocal microscopy

For immunofluorescence experiments, cells plated on coverslips were treated with the indicated siRNA (i.e., NT or S1R) or HA-S1R transfected prior to infection. At 24 hpi, the cells were fixed with 2% paraformaldehyde and permeabilized with 0.3% Tween-20 for 15 min. The samples were blocked using 1% BSA for 1 h at room temperature. The cells were incubated with primary antibody at room temperature for 1 h, followed by 3 washes with 1% BSA. The samples were then stained with AlexaFluor-labelled specific antibodies. The coverslips were mounted using ProLong Gold antifade reagent with DAPI. Images were acquired in SP8 Leica confocal microscope and processed using FIJI (ImageJ) software. For overexpression IF, cells were transfected with HA-S1R prior to infection, followed by the IF protocol.

### Freeze-thaw method

To isolate the intracellular virus [21,22], siRNA-treated Huh-7 cells were infected with DENV for 24 h. The infected cells were washed with PBS and pelleted at 16,000 rpm for 10 min. The subsequent pellet was subjected to 4 freeze-thaw cycles, alternating between liquid nitrogen and water at 37 °C. After the 4 cycles, the sample was pelleted, and the supernatant was collected for focus forming assay and RNA isolation.

### Transmission electron microscopy

For TEM, siRNA (i.e., NT or S1R) -treated cells were infected with DENV for 48 h prior to fixation with 2.5% glutaraldehyde and 2% PFA in 0.1M sodium cacodylate buffer (CB) at 4°C. Post-fixation, the cells were washed with CB and incubated with 1.5% K_4_Fe (CN)_6_ + 2% OsO_4_ in 0.1M CB for 15 minutes. After washing, the cells were stained with 2% uranyl acetate (UA) for 20 minutes, serially dehydrated with ethanol concentrations (30%, 50%, 70%, 80%, 90%, 95%, 100%, 100%) for 5 min each. Following the wash, the samples were incubated with ethanol:epon resin (at a ratio of 2:1,1:1 and 1:2) prior to embedding with 100% resin polymerized at 70 °C for 24 h. Thin sections of the resin were prepared and placed on copper grids and imaged on JEOL JEM 1400 Plus TEM. For negative staining of the extracellular virus, carbon-coated copper grids (300 mesh; Electron Microscopy Sciences, #CF300-CU-50) were glow-discharged for 30 seconds at 10 mA using an EasiGlow system (Pelco). A 3 µL drop of the sample was applied to the grid and allowed to sit for 1 min at room temperature. Excess liquid was blotted off with Whatman 1 filter paper (Cytiva, #1001090). The grid was washed twice with Milli-Q water and once with 0.75% uranyl formate (Electron Microscopy Sciences, # 16984-59-1). The grid was stained for 1 min in a fresh droplet of 0.75% uranyl formate before being subjected to final manual blotting and air drying. Grids were imaged using transmission electron microscope (T12, Tecnai) operated at an acceleration voltage of 120kV. The images were recorded on a Gatan Rio9 CMOS Detector with a pixel size of 2.41 Å.

### *In vitro* furin cleavage

For furin-mediated cleavage of extracellular prM, the supernatant from S1R-depleted cells was collected 48 hpi, concentrated, and incubated in a furin cleavage buffer [20 mM HEPES, 0.1% Triton X-100, 1 mM CaCl_2_, 0.2mM β-mercaptoenthanol (pH 7.5)] containing 2 μl of furin at 25°C for 12 h. The sample was then incubated with 1X sample buffer at 95°C for 15 min before being subjected to SDS-PAGE and Western blotting.

### Sucrose gradient ultracentrifugation

Huh-7 cells transfected with control (i.e., NT) or S1R siRNA for 48 h were infected with DENV for 48 h in 2% FBS media, and the secreted media was collected. The media was concentrated, layered on top of 30–50% continuous sucrose gradient, and the samples centrifuged at 28,000 rpm in a TLA- 100 rotor for 1 h at 4°C on Optima Max-XE Tabletop Ultracentrifuge (Beckman Coulter). 10 fractions (each 20 μL) were collected, incubated with 1X sample buffer, heated at 95°C for 15 min, followed by SDS-PAGE and Western blotting. RNA was extracted with the Qiagen RNeasy kit and subjected to RT-qPCR as above. Stock DENV was fractionated as above.

### Statistical analysis and quantification

All the colocalization quantification was done using FIJI software. Results are shown as mean ± standard deviation (SD) from 3 or more independent experiments. Data sets were statistically analyzed by one-way ANOVA or paired student’s-test in GraphPAD PRISM 10. The significance was determined by p-value, ***p < .0005, **<= 0.005, *<= 0.05. and ns = not significant.

## Supporting information

S1 Fig Additional characterization of DENV in control and S1R-depleted cells (related to Fig 3). **(A)** Additional TEM images of DENV-infected control cells (top row), with the corresponding measurements of the virus diameter shown in the bottom row. **(B)** As in A, except images are from S1R-depleted cells. **(C)** Immunofluorescence staining of Huh-7 cells with viral prM (green) and the *trans* Golgi marker TGN46 (red) infected with DENV (1 MOI) for 24 h. Scale bar: 10 µm **(D)** Quantification of colocalization by Pearsons’ colocalization coefficient. n = 14.
(TIF)

S1 Data Figures and Supplementary file. (PDF)
