# Supplementary material for: The Sigma1 ER membrane receptor promotes structural protein folding and genome packaging of dengue virus
Source: PLoS Pathog. 2026 Jul 16;22(7):e1014347. doi: 10.1371/journal.ppat.1014347 (PMC13374893; doi:10.1371/journal.ppat.1014347)
Supplement: S1 Data — (PDF) [file ppat.1014347.s002.pdf]

Fig 1A

Relative Band intensity (normalized to NT)				
	NT	siS1R	siSCPS1	
sample1	1	0.198195	0.260321	
sample2	1	0.219534	0.088557	
sample3	1	0.105498	0.285687	
sample4	1	0.070212	0.333406	
sample5	1	0.070462	0.243339	

Fig 1B

Extracellular FFU/ml (log10)			
	NT	siS1R	siSCPS1
sample1	4.845098	3.709694	2.574031
sample2	4.875061	3.945099	3.19382
sample3	6.20412	5.190332	4.72632
sample4	6.989005	5.860338	5.574031

Fig 1C

% Cell viability (normalized to NT)			
	NT	siS1R	siSCPS1
sample1	100	96.86459	98.88169
sample2	100	98.95886	98.18652
sample3	100	94.32112	99.22759

Fig 1D

Extracellular FFU/ml (log10) **Rescue**			
	NT	siS1R	siS1R+HA-S1R
sample1	4.852785	3.844068	4.49485
sample2	5.49485	4.49485	5.051153
sample3	5.477121	4.628389	5.419129

Fig 1E

% Cell viability (normalized to NT)		
	HA-mcherry	HA-S1R
sample1	100	96.17767
sample2	100	98.99626
sample3	100	97.06789

Fig 1F

Extracellular FFU/ml (log10) **Inhibitors**			
	DMSO	S1Ra	Haloperidol
sample1	6.273001	4.942008	4.954243
sample2	5.511883	4.544068	4.39794
sample3	6.653213	5.267172	5.079181
sample4	5.778151	4.837273	4.588272

Fig 1G

% Cell viability (normalized to NT)			
	DMSO	S1Ra	Halo
sample1	100	98.48699	94.02686
sample2	100	93.69688	85.52665
sample3	100	96.45731	88.3757

Fig 1H

Normalized % inhibition to (DMSO) (CC50)										
uM(conc)	S1Ra			Average	Std	Halo			Average	Std
DMSO	100	100	100	100		100	100	100		
10	89.50928	90.37576	95.541	91.80868	3.26	77.74412	88.3357	85.04336	83.70773	5.42064
25	89.21087	87.8649	83.03595	86.70391	3.25	58.98431	54.37553	60.11459	57.82481	3.04015
50	65.1204	64.1224	62.09964	63.78081	1.54	54.06556	52.77873	49.73543	52.19324	2.22365
100	36.08045	27.66846	28.27675	30.67522	4.69	33.93798	39.26861	35.8828	36.36313	2.69758

Drug concentration (uM)(IC50)	S1Ra				Haloperidol		
0	100	100	100	100	100	100	100
5	126.087	67.5497	108.5995	76	61.5894	59.9509	64
15	65.2174	46.3576	94.3489	76	21.1921	47.1744	38.6667
40	43.4783	31.7881	37.8378	45.3333	9.2715	32.9238	33.3333
70	28.2609	19.2053	40.7862	37.3333	1.3245	31.4496	28
100	34.7826	12.5828	35.3808	28	0	41.769	28

Fig 1I

intracellular FFU/ml (log10)			
	NT	siS1R	siSPCS1
sample1	4.419129	3.559308	2.559308
sample2	5.258278	4.439333	3.860338
sample3	4.528274	3.750123	2.740363
sample4	5.829304	4.30103	3.511883
sample5	6.896251	5.574031	4.687975

Fig 2A

	Relative viral protein level (normalized to NT)					
Viral Proteins	NT (n=3)			siS1R (n=3)		
prM	1	1	1	0.704515	0.891139	1.219381
Capsid	1	1	1	0.714259	0.94721	0.810123
Envelope	1	1	1	0.92201	0.695414	1.1849
NS1	1	1	1	0.85274	0.853207	1.276286
NS2B	1	1	1	0.917962	0.846058	1.17176
NS4A	1	1	1	0.906591	0.939583	1.466153

Fig 2D

Mean Fluorescence Intensity (MFI)		
	NT	S1R
cell 1	22.4	34.94
cell 2	13.06	12.54
cell 3	16.09	15.45
cell 4	19.77	19.32
cell 5	20.38	18.32
cell 6	23.32	36.49
cell 7	29.31	37.95
cell 8	26.71	21.78

cell 9	26.12	39.11
cell 10	34.44	24.49
cell 11	29.42	27.28
cell 12	27.99	28.77
cell 13	37.66	24.78
cell 14	34.3	36.35
cell 15	25.42	18.97
cell 16	24.92	28.83
cell 17	34.63	20.11

Fig 2E

Relative intracellular RNA level (normalized to NT)			
	NT	siS1R	siSPCS1
Sample1	1	0.925899	0.05528452
Sample2	1	0.779129	0.15759014
Sample3	1	1.232948	0.21645698
Sample4	1	1.214796	0.35989059

Fig 2F

Extracellular viral copy number		
	NT	S1R
Sample1	1.35E+06	1.62E+05
Sample2	9.20E+05	7.54E+05
Sample3	8.18E+05	2.67E+05
Sample4	4.05E+06	2.55E+06

Fig 2H

Number of RO/cells		
	NT	S1R
cell 1	6	9
cell 2	5	4
cell 3	9	9
cell 4	5	6
cell 5	5	5
cell 6	7	5
cell 7	5	4
cell 8	5	3
cell 9	4	4
cell 10	7	5
cell 11	7	5
cell 12	5	6

Fig 3B

Virion Diameter (nm)		
	**NT**	**S1R**
virion 1	50.1	36.2
virion 2	49.3	36.5
virion 3	50.7	39
virion 4	60.3	34.6
virion 5	50.3	33.2
virion 6	45.7	33.4
virion 7	57.3	33.7
virion 8	56.7	40
virion 9	55.4	31.2
virion 10	45.5	30.5
virion 11	51	37.1
virion 12	51.5	32.5
virion 13	48.4	39.8
virion 14	54.8	41.7
virion 15	56.3	34.2
virion 16	53.3	32.3
virion 17	56.9	34.2
virion 18	46	30.8
virion 19	47.4	37.1
virion 20	49.9	32.5
virion 21	54.9	34.2
virion 22	52	35
virion 23	46.7	38
virion 24	52	37.2
virion 25	51	32.2
virion 26	54.9	38.9
virion 27	55.4	36.7
virion 28	50.2	32.3
virion 29	51.9	35.1
virion 30	55.9	35.1
virion 31	54.2	37.8
virion 32	47.6	31.4
virion 33	45.4	31
virion 34	46.4	37.2
virion 35	47	31.9
virion 36	57.6	38.5
virion 37	45.8	36.4
virion 38	45.5	34.9
virion 39	47.9	34.9

Fig 3C

# of virion/cell		
	NT	S1R
Cell 1	11	8
Cell 2	15	18
Cell 3	11	10
Cell 4	12	9
Cell 5	7	14
Cell 6	15	20
Cell 7	15	15
Cell 8	20	10
Cell 9	16	8
Cell 10	13	11
Cell 11	9	9

virion 40	53.5	39.5
virion 41	47.8	34.8
virion 42	56.4	39.3
virion 43	54.3	36.6
virion 44	46.7	35
virion 45	46.6	38.5
virion 46	48.3	33.8
virion 47	54.2	35
virion 48	55.9	36.2
virion 49	49.4	34.2
virion 50	46.3	33.6
virion 51	47.6	31.6
virion 52	49.8	42.8
virion 53	53.3	33.1
virion 54	52	31
virion 55	47.8	34.4
virion 56	53.8	30.7
virion 57	54.9	32
virion 58	51.5	31
virion 59	58.4	35.8
virion 60	50.4	36.6
virion 61	54.4	34.2
virion 62	47	40
virion 63	51.6	32.7
virion 64	47.7	35.5
virion 65	51.6	32.9
virion 66	45.7	37.8
virion 67	43.6	38.2
virion 68	51.4	31.1
virion 69	48.8	34.4
virion 70	46.2	33.3
virion 71	47.7	32.4
virion 72	48.2	32.3
virion 73	54.4	37.4
virion 74	51.5	38.7
virion 75	47.3	33.1
virion 76	53.8	37.6
virion 77	53.4	33.5
virion 78	58.8	31.1
virion 79	52.9	34.1
virion 80	52.5	33.1
virion 81	56.2	36.3
virion 82	49.7	34.9

virion 83	52.5	35.1
virion 84	44.5	34.6
virion 85	51.8	37
virion 86	42.3	34.5
virion 87	59	35.6
virion 88	46.3	35.8
virion 89	45.5	35.3
virion 90	50.1	34.1
virion 91	53.9	32.7
virion 92	48.2	34.7
virion 93	55.8	36.7
virion 94	48	34.7
virion 95	46.7	34.1
virion 96	44.8	37.9
virion 97	46	33
virion 98	43.3	32.3
virion 99	46.2	38.2
virion 100	46.7	37.2
Average	50.64	35.028
Stdev	4.136411	2.665912

Fig 3D

	# of ER assembly sites	
# of virion	NT	S1R
1	19	25
2	20	26
3	10	5
4	6	5
5	4	1

Fig 3F

Pearsons coefficient		
	NT	siS1R
Cell 1	0.346	0.889
Cell 2	0.331	0.804
Cell 3	0.578	0.91
Cell 4	0.567	0.714
Cell 5	0.382	0.802
Cell 6	0.538	0.776
Cell 7	0.44	0.887
Cell 8	0.545	0.81
Cell 9	0.443	0.89
Cell 10	0.46	0.784
Cell 11	0.549	0.898
Cell 12	0.63	0.882
Cell 13	0.55	0.769
Cell 14	0.367	0.783
Cell 15	0.421	0.801
Cell 16	0.429	0.735
Cell 17	0.351	0.747

Cell 18	0.48	0.781
Cell 19	0.346	0.779
Cell 20	0.52	0.877

Fig 4C

Pearsons Coefficient			
	HA-S1R:prM	HA-S1R:NS3	HA-S1R:NS2B
Cell 1	0.825	0.58	0.573
Cell 2	0.854	0.76	0.782
Cell 3	0.896	0.767	0.573
Cell 4	0.886	0.723	0.592
Cell 5	0.901	0.743	0.696
Cell 6	0.916	0.5994	0.643
Cell 7	0.929	0.78	0.737
Cell 8	0.831	0.776	0.793
Cell 9	0.899	0.753	0.708
Cell 10	0.798	0.599	0.807

Fig 4E

Relative viral protein level (normalized to NT) (Detergent soluable)						
Viral protein	NT (n=3)			siS1R (n=3)		
prM	1	1	1	0.208819	0.203257	0.328362
Capsid	1	1	1	0.475937	0.351017	0.373109
Envelope	1	1	1	0.489694	0.480805	0.371017
NS1	1	1	1	0.982347	0.831214	1.172394
NS2B	1	1	1	0.865531	1.12952	0.91
NS4A	1	1	1	0.782889	0.838218	1.00397

Fig 5B

Extracellular virus diameter (nm)		
	NT	S1R
Virion 1	53.86	26.30
Virion 2	49.69	29.85

Virion 3	65.55	28.39
Virion 4	51.77	28.39
Virion 5	52.54	28.18
Virion 6	55.67	31.32
Virion 7	46.45	30.27
Virion 8	45.09	30.90
Virion 9	56.78	33.61
Virion 10	46.14	26.30
Virion 11	46.76	29.23
Virion 12	46.64	38.41
Virion 13	50.94	37.58
Virion 14	49.11	26.72
Virion 15	46.62	27.56
Virion 16	49.13	28.39
Virion 17	46.99	27.35
Virion 18	48.02	29.65
Virion 19	50.73	27.77
Virion 20	49.27	29.23
Virion 21	48.43	39.67
Virion 22	49.27	32.99
Virion 23	47.39	27.56
Virion 24	51.36	25.47
Virion 25	47.60	28.81
Virion 26	49.48	28.39
Virion 27	48.50	29.23
Virion 28	45.66	28.39
Virion 29	51.36	31.32
Virion 30	56.37	38.41
Virion 31	54.20	37.80
Average	50.24	30.43
STDEV	4.25	3.98

Fig 5D

Extracellular RNA-coip (normalized to NT)		
	NT	S1R
Sample1	1	0.188311
Sample2	1	0.436977
Sample3	1	0.51464

Supplementary S1

Pearsons Coefficient		
	NT	S1R
Cell 1	0.325	0.251
Cell 2	0.266	0.108
Cell 3	0.439	0.253
Cell 4	0.187	0.347
Cell 5	0.261	0.305
Cell 6	0.492	0.198
Cell 7	0.442	0.344
Cell 8	0.347	0.156
Cell 9	0.512	0.077
Cell 10	0.225	0.12
Cell 11	0.456	0.211
Cell 12	0.48	0.37
Cell 13	0.429	0.23
Cell 14	0.446	0.145
