# Supplementary material for: The Sigma1 ER membrane receptor promotes structural protein folding and genome packaging of dengue virus
Source: PLoS Pathog. 2026 Jul 16;22(7):e1014347. doi: 10.1371/journal.ppat.1014347 (PMC13374893; doi:10.1371/journal.ppat.1014347)
Supplement: Attachment — Submitted filename: Sarkar_et_al._(Response_to_Reviewers)_auresp_2.docx [file ppat.1014347.s005.docx]

**Reviewer #1:**

**Comment 1:** Additional evidence is needed to establish that the prM immunoprecipitation is isolating virion-associated material and to identify which gradient fraction contains the defective particles. Analysis of viral RNA abundance across the gradient fractions would also help clarify whether the extracellular viral RNA detected in SR1-depleted conditions is secreted within extracellular vesicles, as proposed by the authors (co-fractionation with TSG101), or whether it instead accumulates in higher-density fractions with prM and capsid.

As requested, we performed an additional prM immunoprecipitation (IP) experiment. Specifically, we found that precipitation of intracellular virions by prM IP from control cells pulled down vRNA, as well as the capsid (C) and envelope (E) proteins (**Fig. 5D, right, intracellular),** demonstrating that the prM IP method can isolate the viral structural proteins and genome. Importantly, under S1R KD, precipitation of prM pulled down less vRNA and C (but a similar level of E) when compared to control cells; a lower level of the C protein is expected because the capsid protein associates with vRNA to assist viral packaging. This result demonstrates that genome packaging of intracellular Dengue virus is impaired in S1R-depleted cells, in complete agreement with the finding that less vRNA associates with the extracellular (secreted) virions (**Fig. 5D, left, extracellular**). Together, we believe these results strongly support the hypothesis that S1R plays an important role in genome packaging of Dengue virus.

Also requested, we collected RNA from the sucrose gradient fractions and performed RT-qPCR to measure vRNA abundance across the fractions (**Fig. 5C, bottom graph**). Our new results show that while there is a pool of vRNA in fractions 1-3 that cofractionates with TSG101 in the S1R KD (as well as in control) cells, there is clearly another pool of vRNA in the higher-density fraction (i.e. fraction 5) that cofractionates with prM and capsid proteins under S1R KD. One interpretation of these findings is that under S1R KD, a pool of vRNA is secreted by extracellular vesicles, while another pool accumulates with the aggregated prM/capsid proteins. This is now stated in the Results section.

**Reviewer #2:**

**Comment 1:** To make the proposed claim, the authors should evaluate both capsid and prM association to vRNA inside the cells, that is during the viral assembly process.

Please see response to Reviewer #1, Comment #1 (specifically the first paragraph).

**Comment 2:** The authors did evaluate the impact of a 25 uM treatment with S1Ra and Halo drugs on cell viability (Fig 1G). However, they did not determine their CC50 value as specifically requested. This is of importance to evaluate the selectivity index (CC50/EC50) since the EC50 is between 50 and 76 uM (higher than the concentration tested for viability). Moreover, since S1R regulates assembly, determining the EC50 with FFU assays would be more appropriate than with the % of infection used here.

As requested, in the revised Fig. 1H, we have now calculated the 50% inhibitory concentration (IC_50_) values of S1Ra and Halo based on an FFU assay. The IC_50_ values for S1Ra and Halo are 20.1 uM and 8.3 uM, respectively. We have also included the 50% cytotoxic concentration (CC_50_) values based on an MTS assay. The CC_50_ values for S1Ra and Halo are 77 uM and 48 uM, respectively. (Note that we have added additional data points from the previous version for this calculation). The selectivity index (defined as CC_50_/IC_50_) is 3.83 for S1Ra and 5.78 for Halo.

**Comment 3:** To appropriately compare the efficacy of prM maturation rescue by furin, the authors must show in the same figure/membrane conditions 1,2 and 3.

As requested, we now display conditions 1, 2 and 3 on the same membrane in Fig. 5A (left). Additionally, we quantified the ratio of the prM to pr band intensities in Fig. 5A (bottom graph), which indicates defective cleavage of prM under S1R KD.

**Comment 4:** New Fig 5C is interesting. However, to claim that fractions 1-3 contains assembled particles, infectivity assays should be performed with these fractions. One cannot exclude that fractions 1-3 correspond to extracellular vesicles (since they contain TSG101), free proteins or disassembled particles. In which fractions is the vRNA?

Please see Reviewer #1, Comment 1 (specifically the 2^nd^ paragraph).
