# Supplementary material for: The Sigma1 ER membrane receptor promotes structural protein folding and genome packaging of dengue virus
Source: PLoS Pathog. 2026 Jul 16;22(7):e1014347. doi: 10.1371/journal.ppat.1014347 (PMC13374893; doi:10.1371/journal.ppat.1014347)
Supplement: Attachment — Submitted filename: Sarkar et al. (Response to Reviewers).docx [file ppat.1014347.s004.docx]

**Thank you for the opportunity to revise our manuscript. In this revision, we first address the editor comments, and then the specific points of the three reviewers.**

**I. Editor Comments:**

1. The inconsistency regarding the role of S1R in virus assembly as claimed in the manuscript and the fact that virions are still assembled, but smaller in S1R depleted cells. Therefore, more insights into the composition of these defective virions and how they are forming is required for defining the role of S1R in assembly. The reviewers make a couple of suggestions that address this main critique. Overall, it needs to be clarified whether S1R contributes to assembly of virus maturation.

2. Determining the composition of the smaller virions in S1R depleted cells in comparison to normal virions.

(The response here addresses the first two points.)

As requested, we performed additional biochemical experiments and EM analysis to more thoroughly evaluate the structure and composition of the secreted virions from control (i.e. non-targeting: NT) and S1R knockdown (KD) cells. We put all these results in a new figure (i.e. Fig. 5) and believe these findings have allowed us to better elucidate the role of S1R in promoting folding of DENV structural proteins that impact genome packaging.

First, because the DENV structural proteins became detergent-insoluble under S1R KD (Fig. 4E), we tested whether these misfolded virus proteins are secreted. In the media of control cells, we found prM and its cleaved product pr (Fig. 5A, lane 1). By contrast, only prM was secreted from the S1R-depleted cells (Fig. 5A, lane 2), indicating that prM was not properly cleaved in the infected cells prior to secretion. This result is consistent with our new result showing decreased co-localization of prM with the TGN (where furin-mediated cleavage occurs) under S1R KD (Fig. S1C and S1D). When exogenous furin was incubated with the media from S1R-depleted cells, pr is generated (Fig. 5A, lane 3), demonstrating that secreted prM is competent for furin-cleavage. Additionally, we found that the E protein was efficiently secreted under S1R KD when compared to control cells (Fig. 5A, compare lane 5 to 4 in top blot), while secretion of the C protein modestly decreased (Fig. 5A, compare lane 5 to 4 in second blot). Thus, despite the DENV structural proteins all becoming misfolded under S1R KD (Fig. 4E), secretion of prM and E remains unimpeded with C protein secretion being moderately affected.

Second, we determined whether DENV particles are assembled when they are secreted by S1R-depleted cells. Again, by EM analysis, we found that the DENV particles secreted from S1R-depleted cells can be assembled but are smaller when compared to control cells (Fig. 5B, compare examples in bottom to top row), with virus released under S1R KD measuring approximately 30 nm in diameter while those from control cells are 50 nm in diameter (Fig. 5B, right graph). These measurements are similar to those observed within the infected cells (Fig. 3A and 3B).

Third, we used sucrose gradient analysis to further characterize the gross structural state of the virions secreted from control and S1R-depleted cells; we used stock DENV as a positive control because this virus should be assembled. The majority of the stock DENV—as assessed by the fractionation pattern of prM/pr, E, and C proteins (Fig. 5C, top three blots, dotted line box)—were detected in fractions 1-3, indicating that these fractions harbor assembled virions. Importantly, the majority of the C and cleaved prM (i.e. pr) proteins, as well as some of the E protein, secreted from control cells were found in fractions 1-3 (Fig. 5C, blots 4-6). Thus, DENV particles in these fractions represent assembled virus.

By contrast, the fractionation pattern of the C and prM structural proteins secreted from S1R KD cells was different. Specifically, low level of the C protein distributed across fractions 1-6, with accumulation in fraction 5 (Fig. 5C, 10^th^ blot), and a low level of the prM protein also distributed across fractions 1-6 (Fig. 5C, 8^th^ blot); no difference was observed in the fractionation pattern of the E protein (Fig. 5C, compare 9^th^ to 5^th^ blot). The broad distribution of the C and prM proteins across more fractions suggests that virions secreted from S1R KD cells exist in a conformation distinct from the control virus, consistent with the EM findings (Fig. 5B). Distribution of the C and prM proteins to denser sucrose fractions (i.e. fractions 4-6) under S1R KD may be explained by their propensity to aggregate because they are detergent-insoluble.

Fourth, to explain the decreased in size of the secreted virus under S1R KD, we performed coIP experiments. Strikingly, we found that IP of prM pulled down less vRNA in virus secreted from S1R-depleted cells when compared to control cells (Fig. 5D, top graph), whereas the level of E protein that co-precipitated with prM was unaffected (Fig. 5D, compare lane 2 to 1 in top blot); we did not detect the C protein in the precipitated material even in the control virus, consistent with the observation that prM does not directly bind to C (**PMID: 14528291, 12773377).** Because DENV particles devoid of the RNA genome are reported to be smaller and approximately 30-35 nm in diameter (**PMID: 29609659, 28956764, 13678860**), one possible explanation for the decreased size of virions secreted from S1R-depleted cells is that the defective virus represents an empty particle without the genome (as astutely noted by a reviewer). Thus, S1R-dependent folding of the virus structural proteins likely plays a role in enabling the efficient packaging of the vRNA.

We note that the level of extracellular vRNA secreted from S1R-depleted cells did not decrease (Fig. 2F). This could be because the great majority of the extracellular vRNA are not packaged with the virus and are instead secreted separately from the virus. This possibility is consistent with presence of extracellular vesicles (EVs) —as indicated by the marker TSG101 in the secreted media of both control and S1R KD cells (Fig. 5C, 7^th^ and 11^th^ blots) —which have been reported to mediate secretion of unpackaged DENV vRNA (**PMID: 38863700**).

3. Corroborating the S1R-prM interaction.

As requested, we added additional data to elucidate the S1R-prM interaction. First, in Fig. 4A (lanes 5-8), we show that the anti-prM antibody does not pulldown HA-tagged S1R (HA-S1R) in uninfected cells, indicating that presence of prM in infected cells is required for the anti-prM antibody to precipitate HA-S1R. This was specifically requested by reviewer 2. Second, in Figure 4A (lanes 9-12), we show that prM interacts with endogenous S1R, as requested by reviewer 2. This result shows that the prM-S1R interaction is unlikely an overexpression artifact. Third, in Fig. 4A (lanes 13-16), we performed a reverse coIP experiment using extracts from infected cells and found that IP of HA-S1R did not pull down prM (or C). This suggests that the majority of S1R does not interact with prM, but instead only a small pool of S1R binds to the prM structural protein during infection. (Because of this finding, we did not carefully evaluate whether the E protein co-precipitated with HA-S1R).

4. Determining the effect of S1R depletion on the solubility of all structural proteins and the assembled defective virions.

As requested, we now show data for all DENV structural proteins during infection. Specifically, we found that while expression of the E protein was unaffected in S1R-depleted cells [Fig. 2A (+quantification)], this structural protein was also resistant to detergent extraction under S1R KD [Fig. 4E (+quantification)]. Additionally, we further evaluated the detergent solubility of transfected prM and E proteins (Fig. 4F), as suggested by a reviewer. Again, while there is only a modest decrease in the expression level of prM and E proteins in S1R-depleted cells (Fig. 4F, 5^th^ and 6^th^ blots, compare lane 2 to 1), both of these proteins cannot be efficiently extracted by detergent under S1R KD (Fig. 4F, 1^st^ and 2^nd^ blots, compare lane 2 to 1). Hence, the chaperone activity of S1R on the DENV structural proteins occurs not only during infection, but also during transfection of the proteins.

5. Determining the impact of knock-downs and drug treatments on cell viability in the various assays.

As requested, we now show data on the effect of S1R KD, chemical inhibition of S1R, and expression of S1R, on cell viability in Figs. 1C, E, and G. We found that in none of these instances was cell viability compromised.

**Part II – Major Issues: Key Experiments Required for Acceptance**
Please use this section to detail the key new experiments or modifications of existing experiments that should be absolutely required to validate study conclusions.

Generally, there should be no more than 3 such required experiments or major modifications for a "Major Revision" recommendation. If more than 3 experiments are necessary to validate the study conclusions, then you are encouraged to recommend "Reject".

**Reviewer #1**:

**Comment 1:** In Figure 3, the authors show that virion size is smaller in S1R depleted cells. It is not clear how this observation is consistent with the results showing that there is a decrease in prM cleavage, since immature particles are larger in size than mature particles. The observed particle size parallels observations for virus-like particles that lack viral RNA, but this would not be consistent with data in Figure 2E showing that RNA section is not altered.

A. The authors should make an effort to determine how the composition of the smaller virions in S1R-depleted cells differs from normal virions.

Please see response to Editor comments 1+2 above.

B. The authors should also show quantification of the number of virions per cell to evaluate if there is an accumulation of virions inside the cell.

As requested, our new EM analysis revealed that there is no increase in the number of virions per cell under S1R KD when compared to control (Fig. 3C), suggesting that there is unlikely any accumulation of the virions inside the cell under the compromised condition. This is consistent with the new finding that the level of the DENV structural proteins prM and E in the secreted media did not significantly decrease under S1R KD (Fig. 5A).

We note that under S1R KD, confocal imaging analysis revealed that co-localization of prM with the ER assembly site marker ATL3 increased (Fig. 3E/F), while co-localization of prM with the *trans* Golgi network (TGN) marker TGN46 decreased (Supplementary Figure 1C/D), even though the total number of virions in S1R KD cells did not change.

**Comment 2:** The manuscript would benefit from further evaluation of extracellular viral particles secreted from S1R depleted cells. In figure 3B, the authors show that all intracellular particles in S1R depleted cells are defective, with no particles having the proper size. This observation opens questions as to why they do not observe more than a 1-log decrease in virus titers and if the secreted virions are also defective. It is possible that the smaller particles are not secreted.

A. Negative stain EM should be done on the secreted virions to determine if they are also smaller in size.

As requested, we have now performed negative-stain EM and found that the secreted virions from S1R KD cells are smaller when compared to secreted virions from control cells (Fig. 5B), similar to the intracellular virions in S1R-depleted cells (Fig. 3A/B).

B. The secreted virus should also be treated with exogenous furin to determine if the defect is specifically in maturation or if there is a different defect.

As requested, we treated the defected secreted virions from S1R KD cells with exogenous furin and found that prM is cleaved to generate pr (Fig. 5A, lane 3), suggesting that the prM of the secreted defective virions can be cleaved but is inefficiently cleaved in cells because of mis-trafficking to the TGN (Supplementary Fig. 1C/D).

C. Western blotting for capsid and envelope could also be used to determine the presence of other structural proteins.

Yes, this was done. Please see response to Editor comments 1+2 above.

**Comment 3:** The authors suggest that S1R functions as a chaperone to decrease structural protein aggregation, which allows for assembly. It is unclear from the experiments whether individual proteins are less soluble or if the assembled defective particles are less soluble.

A. Is envelope solubility also decreased in S1R siRNA treated cells?

As requested, we now show that the E protein made during infection was difficult to extract by detergent under S1R KD, similar to the prM and C proteins (Fig. 4E; quantified in right graph).

B. If the solubility of all structural proteins is altered, it would suggest that the smaller virions are less soluble and not the individual proteins. This could also be tested by expression of individual structural proteins in S1R depleted cells outside the context of infection.

As requested, we evaluated the detergent solubility of transfected prM and E proteins (Fig. 4F). Again, while there is only a modest decrease in the expression level of prM and E proteins in S1R-depleted cells (Fig. 4F, 5^th^ and 6^th^ blots, compare lane 2 to 1), both of these proteins cannot be efficiently extracted by detergent under S1R KD (Fig. 4F, 1^st^ and 2^nd^ blots, compare lane 2 to 1). Hence, the chaperone activity of S1R on the DENV structural proteins occurs not only during infection, but also during transfection of the proteins as well.

**Reviewer #2**:

**Comment 1:** The molecular mechanism behind S1R role in DENV production remains unclear based on the data. The authors claims that this host factor regulates particle assembly based on the fact that intracellular virions are smaller in diameter as seen by transmission electron microscopy (Fig 2G-H). However, extracellular prM shows maturation defects (Fig 4F) while extracellular prM/pr (Fig 4F) and RNA levels (Fig 2F) remain unchanged, a phenotype contrasting with the expectations of a potential assembly defect. This implies that S1R might influence the specific specificity instead of assembly as interpreted by the authors. Both possibilities are in line with a decrease in intracellular infectivity (Fig 1E). This must be addressed.

Please see response to Editor comments 1+2 above.

To reiterate, we performed EM analysis on the secreted virus and found that the secreted virions are smaller under S1R KD (Fig. 5B; see quantification in the right graph), similar to the smaller virus found in S1R KD cells (Fig. 3A/B). Additionally, we performed coIP experiments to study interaction between the virus structural protein and the vRNA in the secreted virus. Strikingly, we found that IP of prM pulled down less vRNA in virus secreted from S1R-depleted cells when compared to control cells (Fig. 5D, top graph), whereas the level of E protein that co-precipitated with prM was uneffected (Fig. 5D, compare lane 2 to 1 in top blot); we did not observe co-precipitation of the C protein with prM even in the control virus, consistent with the observation that prM does not directly bind to C (**PMID: 14528291, 12773377).** Because DENV particles devoid of the RNA genome are reported to be smaller and approximately 30-35 nm in diameter (**PMID: 29609659, 28956764, 13678860**), one possible explanation for the decreased size of virions secreted from S1R-depleted cells is that the defective virus represents an empty particle without the genome (as astutely noted by a reviewer). Thus, S1R-dependent folding of the virus structural proteins (Fig. 4E) likely plays a role in enabling the efficient packaging of the vRNA.

**Comment 2:** Is the stoichiometry of virion components changed upon knockdown? Are Env and Capsid efficiently incorporated in particles and released?

Please see response to Editor comments 1+2 above.

**Comment 3:** In EM analysis, the authors should quantify the abundance of virus bags (ER cisternae containing multiple virions) and the number of virions per bag, which might provide insightful information to refine their model. Most importantly, to assess, why prM shows maturation defects under knockdown conditions, the authors must analyze prM localization in the trans Golgi network which, if decreased, could explain an impairment in its furin-mediated cleavage. This would also address whether there is a trafficking alteration.

As requested, in the new Fig. 3D, we quantified virions in dilated ER cisternae (“virus bags”) that are generally thought to represent particle assembly sites, as well as the number of virions per assembly site, and found only small differences between control and S1R KD cells. For instance, in control cells, there were approximately 20 ER sites that harbor either 1 or 2 viral particles, while in S1R KD cells, there were approximately 23 ER sites that harbor either 1 or 2 viral particles.

Additionally, we performed co-localization studies and found that depletion of S1R decreased prM-TGN46 colocalization (Supplementary Fig. 1C; quantified in Supplementary Fig. 1D). As this reviewer correctly pointed out, this likely explains the impairment in furin-mediated cleavage, and raises the possibility that under S1R KD, the virions experienced altered trafficking.

**Comment 4:** Finally, the authors conclude that S1R depletion impact prM solubility but in contrast, its release does not seem to be altered under the same knockdown conditions. How do the authors reconcile these two observations? Could it be release within extracellular vesicles?

Although we found that the detergent-insoluble prM can still be secreted under S1R KD (Fig. 5A), whether its secretion follows the conventional pathway is unclear, especially given that prM in S1R KD cells have decreased TGN colocalization (see Comment 6 above). Of note, we found presence of the extracellular vesicle marker TSG101 in the secreted media of control and S1R KD cells (Fig. 5C, 7^th^ and 11^th^ blots), raising the possibility that the defective DENV could be released within EVs.

**Comment 5:** In addition, since prM colocalizes with HA-S1R, they conclude an association with the assembly complexes. The existence of a prM/S1R complex is supported by co-IP assays. However, critical controls are missing in both sets of experiments. Considering the broad ER localization of both proteins, the observed colocalization in confocal microscopy is expected and might not reflect a bona fide interaction between the two proteins, but instead, a similar organelle distribution. Thus, the authors must repeat the analysis with antibodies against ER-associated viral nonstructural proteins labeling the replication complexes. If the hypothesis of the authors is true, the Pearson’s coefficient is expected to be lower in such an analysis.

As requested, we now show HA-S1R colocalization with the NS3/NS2B ER-associated viral nonstructural proteins that label the replication complex and indeed found a decrease in Pearson’s coefficient (Fig. 4B; quantified in 4C).

**Comment 6:** The anti-prM IP assay of Fig 4A lacks the uninfected control. This condition is important to demonstrate that HA-S1R is not pulled down non-specifically with anti-prM antibodies. Without this specificity control, the data are unconclusive. In addition, Envelop and Capsid should be assessed to determine whether S1R associate with the whole particle or instead only with prM specifically. Finally, since the anti-S1R is available (Fig 1), the demonstration of an interaction between prM and the endogenous S1R would strengthen the conclusion.

Please see response to Editor comment 3.

**Comment 7:** In Fig 1A-C: What is the impact of S1R knockdown and overexpression on cell viability and growth? This must be shown to rule out that the observed viral phenotypes are due to off-target effect or a decreased abundance of virion-producing cells. The same applies for S1Ra and Halo drug treatments. In addition, the EC50 and CC50 values should be determined for these drugs so that the readers appreciate the potency and the selectivity of these S1R inhibitors.

Please see response to Editor comment 5.

Additionally, in Fig. 1H, we show the graph and IC50 values of S1Ra and Halo.

**Reviewer #3**:

**Comment** **1**: The authors demonstrate a strong effect on the morphology of the virions within the cell. These do not appear to be immature virions with uncleaved prM as these are expected to be larger rather than smaller than mature virions where prM is cleaved. Some more characterization of these particles would help to better understand this intriguing observation. The authors already show that S1R depletion results in prM cleavage defects in the medium but it is unclear if this prM is incorporated in viral particles or somehow is independently secreted. qPCR analysis indicates that secreted viral RNA levels are unaffected suggesting that viral particles are being assembled and secreted. Do these secreted viral particles contain less Envelope protein and/or Capsid? This could be determined using western blotting on concentrated medium as the authors performed using prM antibodies. Ideally this would be done after density gradient ultracentrifugation to separate the particles, but I could understand that this falls outside of the scope of the current manuscript.

Please see response to Editor’s comments 1+2 above.

**Comment 2**. The cleavage defect of prM secreted in the medium is intriguing. If S1R acts as a chaperone, can the cleavage defect be rescued by lowering the temperature during viral assembly and/or can Furin overexpression rescue the cleavage defect?

As requested, we treated the defected secreted virions from S1R KD cells with exogenous furin and found that the prM is cleaved (Fig. 5A, lane 3), indicating that the prM of the secreted defective virions can be cleaved but is inefficiently cleaved in cells because of inefficient trafficking to the TGN (Supplementary Fig. 1C/D).

**Comment 3**. The reduction in detergent solubility is interpreted to reflect misfolding leading to non-soluble protein aggregates. Do the authors see an increase in non-soluble protein aggregates upon S1R KD to more directly show this?

We attempted a “sequential” detergent extraction experiment, first using a RIPA buffer (where we observe a decrease in DENV structural protein extraction due to depletion of S1R) and then using a 2% SDS buffer to determine if we can observe an increase in the level of the structural proteins under S1R KD. Unfortunately, we were NOT able to observe any protein signal in the SDS step, precluding us from directly observing the increase in the non-soluble proteins.

**Comment 4**. The authors shown that upon infection of cells overexpressing HA epitope tagged S1R, pulldown with prM leads to detectable HA-S1R. Did the authors perform the reciprocal pulldown using HA beads and was prM the only protein that co-immunoprecipitated? prM interacts with Envelope and is a chaperone for Envelope.

Please see response to Editor comment 3 in which we better elucidated the S1R-prM interaction.

**Part III – Minor Issues: Editorial and Data Presentation Modifications**
Please use this section for editorial suggestions as well as relatively minor modifications of existing data that would enhance clarity.

Reviewer #1:

1. What is the impact of S1R depletion on cell growth and viability? Similarly, do the S1R drugs have toxicity?

Please see response to Editor comment 5.

2. Does envelope protein interaction with prM decrease in S1R siRNA treated cells?

Please see response to Editor comments 1+2 above (i.e. third point).

3. Quantification of Vps should be done per cell rather than per field of view.

Thank you. This has been updated in Fig. 2H.

Reviewer #2:

1. S1R seems not to be the official gene name for the protein of interest (i.e. SIGMAR1), which is suggested to be used throughout the manuscript.

We have used S1R in our previous publications (PMID: 39128005) and prefer to be consistent with our work.

2. Line 52: Flavivirus is not a virus family. DENV belongs to the Orthoflavivirus genus within the Flaviviridae family. This must be correct.

Thank you for this correction. This has been updated.

3. Fig 1A-C: What is the impact of S1R knockdown and overexpression on cell viability? This must be shown to rule out that the viral phenotypes are not due to off-target effect or a decreased abundance of virion-producing cells.

Please see response to Editor comment 5.

4. Fig 1C: Western blots showing the knockdown and overexpression efficiency must be shown.

We apologize for this but we used the same system previously (PMID: 39128005).

5. Fig 1G: Micrographs are shown in duplicate. It is suggested that the authors keep only one image per condition.

As suggested, we kept just one image per condition and this has been updated in Fig. 2G.

6. Line 209: In contrast to the conclusion drawn by the authors, NS3 seems to be detected in the control anti-prM IP (HA-mCherry). This signal disappears when S1R is overexpressed. Is this phenotype reproducible and relevant for S1R function?

We believe this is simply an inadvertent smudge in the gel (and not the presence of any real band).

7. Line 156, Fig 2C: The percentage of dsRNA-containing cells is not indicative of the extent of viral RNA replication as concluded by the authors, but instead, simply reflect the proportion of infected cells (i.e., entry or spread depending on the time point post-infection). Instead, the authors should quantify the abundance of dsRNA puncta or the mean fluorescence per cells to be able to draw a conclusion on viral replication.

In Fig. 2D, we have quantified the mean fluorescence intensity/cell, as requested.

8. Fig 2B: A replication kinetics (1-3 days) following the siRNA transfection of the DENV subgenomic replicon stable cell line would be informative. A negative control disrupting replication (e.g., antiviral drug treatment) should be included. Unless this was missed, no information is provided on how this cell line was generated.

We previously characterized this cell line, as published in PMID: 31067454.

9. Is the S1R dependency specific to DENV? If possible, the knockdown replication assays should be repeated with other flaviviruses and an unrelated RNA virus.

This is a great question. We are evaluating whether other flaviviruses (as well as other RNA viruses) depend on S1R for infection, but we hope this reviewer agrees that these proposed experiments are beyond the scope of the present manuscript.

10. What is the diameter of released viral particles? Are the smaller virions observed inside the cell released?

Please see Fig. 5B, quantification.

Reviewer #3:

1. S1R has a role in the formation of cholesterol rich microdomains in the ER and flavivirus prM uses cholesterol for assembly. Can the authors speculate in the discussion whether this may be relevant for the phenotype.

In the Discussion, we have added a brief discussion on the role of S1R in formation of cholesterol-rich microdomains in the ER and how this might impact prM’s dependence on cholesterol for assembly.

2. In the viral production assays measuring FFU’s there is a wide variance (e.g. in 1B). Even for the NT, there can be a 100-fold difference. Are these experiments biological replicated and are the samples paired?

Samples in Fig. 1B, D, F, and I are paired.

3. Figure 2D the y-axis states “normalized to NT” this may be a typo.

The Y-axis in Fig. 2D has been updated to mean fluorescence intensity/cell (arbitrary units), as requested by reviewer 2.
