# Supplementary material for: The Sigma1 ER membrane receptor promotes structural protein folding and genome packaging of dengue virus
Source: PLoS Pathog. 2026 Jul 16;22(7):e1014347. doi: 10.1371/journal.ppat.1014347 (PMC13374893; doi:10.1371/journal.ppat.1014347)
Supplement: Peer review history

# Peer review: Decision Letter 0

**DOI:** 10.1371/journal.ppat.1014347.r001

**Authors:** Alexander Gorbalenya, Ralf Bartenschlager

3 Dec 2025

-->PPATHOGENS-D-25-02764

The Sigma1 ER membrane receptor promotes the post-replication assembly of Dengue virus

PLOS Pathogens

Dear Dr. Tsai,

Thank you for submitting your manuscript to PLOS Pathogens. After careful consideration, we feel that it has merit but does not yet meet PLOS Pathogens's publication criteria as it currently stands. Therefore, we invite you to submit a revised version of the manuscript that addresses all the points raised during the review process.

Please submit your revised manuscript by Feb 01 2026 11:59PM. If you will need more time than this to complete your revisions, please reply to this message or contact the journal office at plospathogens@plos.org. When you're ready to submit your revision, log on to https://www.editorialmanager.com/ppathogens/ and select the 'Submissions Needing Revision' folder to locate your manuscript file.

Please include the following items when submitting your revised manuscript:

* A rebuttal letter that responds to each point raised by the editor and reviewer(s). You should upload this letter as a separate file labeled 'Response to Reviewers'. This file does not need to include responses to any formatting updates and technical items listed in the 'Journal Requirements' section below.

* A marked-up copy of your manuscript that highlights changes made to the original version. You should upload this as a separate file labeled 'Revised Manuscript with Track Changes'.

* An unmarked version of your revised paper without tracked changes. You should upload this as a separate file labeled 'Manuscript'.

If you would like to make changes to your financial disclosure, competing interests statement, or data availability statement, please make these updates within the submission form at the time of resubmission. Guidelines for resubmitting your figure files are available below the reviewer comments at the end of this letter.

We look forward to receiving your revised manuscript.

Kind regards,

Ralf Bartenschlager, PhD

Guest Editor

PLOS Pathogens

Alexander Gorbalenya

Section Editor

PLOS Pathogens

Sumita Bhaduri-McIntosh

Editor-in-Chief

PLOS Pathogens

orcid.org/0000-0003-2946-9497

Michael Malim

Editor-in-Chief

PLOS Pathogens

orcid.org/0000-0002-7699-2064

**Additional Editor Comments :**

All three reviewers consider the finding on the role of S1R in DENV assembly interesting and novel. At the same time, they raise a number of concerns regarding the mechanistic aspect by which S1R supports assembly or virion maturation as well as inconsistencies between the conclusions and the data. The most important ones to be addressed are:

1. The inconsistency regarding the role of S1R in virus assembly as claimed in the manuscript and the fact that virions are still assembled, but smaller in S1R depleted cells. Therefore, more insights into the composition of these defective virions and how they are forming is required for defining the role of S1R in assembly. The reviewers make a couple of suggestions that address this main critique. Overall, it needs to be clarified whether S1R contributes to assembly of virus maturation.

2. Determining the composition of the smaller virions in S1R depleted cells in comparison to normal virions.

3. Corroborating the S1R-prM interaction.

4. Determining the effect of S1R depletion on the solubility of all structural proteins and the assembled defective virions.

5. Determining the impact of knock-downs and drug treatments on cell viability in the various assays.

There are additional points raised by the reviewers that have to be addressed, before the manuscript can be re-considered for publication.

**Journal Requirements:**

1) Please ensure that the CRediT author contributions listed for every co-author are completed accurately and in full.

At this stage, the following Authors/Authors require contributions: Riya Sarkar, Andrew W. Tai, and Billy Tsai. Please ensure that the full contributions of each author are acknowledged in the "Add/Edit/Remove Authors" section of our submission form.

The list of CRediT author contributions may be found here: https://journals.plos.org/plospathogens/s/authorship#loc-author-contributions

2) We do not publish any copyright or trademark symbols that usually accompany proprietary names, eg ©,  ®, or TM  (e.g. next to drug or reagent names). Therefore please remove all instances of trademark/copyright symbols throughout the text, including:

- ® on pages: 16, and 18

- TM on pages: 16, and 17.

3) Your manuscript is missing the following section: Abstract.  Please ensure all required sections are present and in the correct order. Make sure section heading levels are clearly indicated in the manuscript text, and limit sub-sections to 3 heading levels. An outline of the required sections can be consulted in our submission guidelines here:

https://journals.plos.org/plospathogens/s/submission-guidelines#loc-parts-of-a-submission

4) We note that your Data Availability Statement is currently as follows: "All relevant data are within the manuscript and its Supporting Information files.". Please confirm at this time whether or not your submission contains all raw data required to replicate the results of your study. Authors must share the “minimal data set” for their submission. PLOS defines the minimal data set to consist of the data required to replicate all study findings reported in the article, as well as related metadata and methods (https://journals.plos.org/plosone/s/data-availability#loc-minimal-data-set-definition).

For example, authors should submit the following data:

1) The values behind the means, standard deviations and other measures reported;

2) The values used to build graphs;

3) The points extracted from images for analysis..

Authors do not need to submit their entire data set if only a portion of the data was used in the reported study.

If your submission does not contain these data, please either upload them as Supporting Information files or deposit them to a stable, public repository and provide us with the relevant URLs, DOIs, or accession numbers. For a list of recommended repositories, please see https://journals.plos.org/plosone/s/recommended-repositories.

If there are ethical or legal restrictions on sharing a de-identified data set, please explain them in detail (e.g., data contain potentially sensitive information, data are owned by a third-party organization, etc.) and who has imposed them (e.g., an ethics committee). Please also provide contact information for a data access committee, ethics committee, or other institutional body to which data requests may be sent. If data are owned by a third party, please indicate how others may request data access.

5) Please revise your current Competing Interest statement to the standard "The authors have declared that no competing interests exist."

Note: If the reviewer comments include a recommendation to cite specific previously published works, please review and evaluate these publications to determine whether they are relevant and should be cited. There is no requirement to cite these works unless the editor has indicated otherwise.

**Reviewers' Comments:**

Reviewer's Responses to Questions

**Part I - Summary**

Please use this section to discuss strengths/weaknesses of study, novelty/significance, general execution and scholarship.

Reviewer #1: The manuscript by Sarkar and colleagues describes a role for the EM membrane receptor Sigma1 (S1R) in supporting DENV assembly. The authors demonstrate that depletion of S1R results in a robust decrease in viral titers, with minimal effects on the percentage of infected cells, viral protein production, or genome replication. They also show that S1R depletion alters the size of DENV virions within cells. Finally, they demonstrate an association between S1R, and the viral prM protein and link S1R depletion to the solubility of viral structural proteins.

The phenotypes shown are robust, and the results provide a compelling argument that S1R supports DENV assembly. This is an interesting finding with the potential to significantly impact our understanding of how host proteins contribute to DENV assembly. However, how S1R contributes to assembly is still unclear. The major concern with the current manuscript is that the conclusions about the role of S1R in virus assembly are not consistent with the observation that virions are still assembled, but smaller in S1R depleted cells. The authors claim that S1R chaperone activity is important for maintaining structural protein solubility, which in turn promotes assembly and maturation. It is not clear how this conclusion is consistent with the observation that smaller virions are still formed in S1R knockdown cells. Understanding more about the biology and composition of these defective virions is important for defining the role of S1R in assembly and supporting the conclusions.

Reviewer #2: In this study, Sarkar and colleagues study the role of Sigma 1 ER membrane receptor (S1R) in the life cycle of dengue virus (DENV). They show that the knockdown of S1R decreases the overall replication of DENV. Further characterization demonstrates that viral replication and protein expression are not impacted by S1R knockdown. Instead S1R regulates the production of infectious particles. Electron microscopy data show that assembled virions are smaller in size while released particles seem more immature (and presumably less infectious) in terms of prM cleavage. Based on co-immunoprecipation (co-IP) and confocal imaging data, the authors conclude that S1R interacts with prM.

The authors convincingly demonstrate that S1R regulate the production of infectious viral particle (and not translation and replication). However, the molecular mechanism behind this regulation remains unclear and some interpretations are not supported by the data, which sometimes lack proper controls. More characterization of S1R role in DENV replication is needed to support and refine the model proposed by the authors.

Reviewer #3: This manuscript characterizes the role of Sigma-1 ER membrane receptor (S1R) in the life cycle of dengue virus infection. Using a knockdown (KD) approach, the authors demonstrate that S1R acts after RNA replication and does not influence polyprotein expression or the formation of replication vesicles. Strikingly, using negative stain electron microscopy they find that intracellular viral particles are significantly smaller upon S1R KD. They demonstrate that PrM binds to overexpressed S1R and that there is a defect in cleavage of prM released into the medium in DENV infected S1R KD cells. The experiments are well-controlled and shed light on host factors important for viral assembly. Overall, the manuscript describes a novel role of an ER-resident chaperone specifically in generation of infectious viral particles significantly contributing to our knowledge on virus-host interactions of the medically important DENV. I have noted few points that could strengthen the manuscript.

**********

**Part II – Major Issues: Key Experiments Required for Acceptance**

Please use this section to detail the key new experiments or modifications of existing experiments that should be absolutely required to validate study conclusions.

Generally, there should be no more than 3 such required experiments or major modifications for a "Major Revision" recommendation. If more than 3 experiments are necessary to validate the study conclusions, then you are encouraged to recommend "Reject".

Reviewer #1: •In Figure 3, the authors show that virion size is smaller in S1R depleted cells. It is not clear how this observation is consistent with the results showing that there is a decrease in prM cleavage, since immature particles are larger in size than mature particles. The observed particle size parallels observations for virus-like particles that lack viral RNA, but this would not be consistent with data in Figure 2E showing that RNA section is not altered. The authors should make an effort to determine how the composition of the smaller virions in S1R depleted cells differs from normal virions. The authors should also show quantification of the number of virions per cell to evaluate if there is an accumulation of virions inside the cell.

•The manuscript would benefit from further evaluation of extracellular viral particles secreted from S1R depleted cells. In figure 3B, the authors show that all intracellular particles in S1R depleted cells are defective, with no particles having the proper size. This observation opens questions as to why they do not observe more than a 1-log decrease in virus titers and if the secreted virions are also defective. It is possible that the smaller particles are not secreted. Negative stain EM should be done on the secreted virions to determine if they are also smaller in size. The secreted virus should also be treated with exogenous furin to determine if the defect is specifically in maturation or if there is a different defect. Western blotting for capsid and envelope could also be used to determine the presence of other structural proteins.

•The authors suggest that S1R functions as a chaperone to decrease structural protein aggregation, which allows for assembly. It is unclear from the experiments whether individual proteins are less soluble or if the assembled defective particles are less soluble. Is envelope solubility also decreased in S1R siRNA treated cells? If the solubility of all structural proteins is altered, it would suggest that the smaller virions are less soluble and not the individual proteins. This could also be tested by expression of individual structural proteins in S1R depleted cells outside the context of infection.

Reviewer #2: The molecular mechanism behind S1R role in DENV production remains unclear based on the data. The authors claims that this host factor regulates particle assembly based on the fact that intracellular virions are smaller in diameter as seen by transmission electron microscopy (Fig 2G-H). However, extracellular prM shows maturation defects (Fig 4F) while extracellular prM/pr (Fig 4F) and RNA levels (Fig 2F) remain unchanged, a phenotype contrasting with the expectations of a potential assembly defect. This implies that S1R might influence the specific specificity instead of assembly as interpreted by the authors. Both possibilities are in line with a decrease in intracellular infectivity (Fig 1E). This must be addressed. Is the stoichiometry of virion components changed upon knockdown? Are Env and Capsid efficiently incorporated in particles and released? In EM analysis, the authors should quantify the abundance of virus bags (ER cisternae containing multiple virions) and the number of virions per bag, which might provide insightful information to refine their model. Most importantly, to assess, why prM shows maturation defects under knockdown conditions, the authors must analyze prM localization in the trans Golgi network which, if decreased, could explain an impairment in its furin-mediated cleavage. This would also address whether there is a trafficking alteration. Finally, the authors conclude that S1R depletion impact prM solubility but in contrast, its release does not seem to be altered under the same knockdown conditions. How do the authors reconcile these two observations? Could it be release within extracellular vesicles?

In addition, since prM colocalizes with HA-S1R, they conclude an association with the assembly complexes. The existence of a prM/S1R complex is supported by co-IP assays. However, critical controls are missing in both sets of experiments. Considering the broad ER localization of both proteins, the observed colocalization in confocal microscopy is expected and might not reflect a bona fide interaction between the two proteins, but instead, a similar organelle distribution. Thus, the authors must repeat the analysis with antibodies against ER-associated viral nonstructural proteins labeling the replication complexes. If the hypothesis of the authors is true, the Pearson’s coefficient is expected to be lower in such an analysis.

The anti-prM IP assay of Fig 4A lacks the uninfected control. This condition is important to demonstrate that HA-S1R is not pulled down non-specifically with anti-prM antibodies. Without this specificity control, the data are unconclusive. In addition, Envelop and Capsid should be assessed to determine whether S1R associate with the whole particle or instead only with prM specifically. Finally, since the anti-S1R is available (Fig 1), the demonstration of an interaction between prM and the endogenous S1R would strengthen the conclusion.

In Fig 1A-C: What is the impact of S1R knockdown and overexpression on cell viability and growth? This must be shown to rule out that the observed viral phenotypes are due to off-target effect or a decreased abundance of virion-producing cells. The same applies for S1Ra and Halo drug treatments. In addition, the EC50 and CC50 values should be determined for these drugs so that the readers appreciate the potency and the selectivity of these S1R inhibitors.

Reviewer #3: 1. The authors demonstrate a strong effect on the morphology of the virions within the cell. These do not appear to be immature virions with uncleaved prM as these are expected to be larger rather than smaller than mature virions where prM is cleaved. Some more characterization of these particles would help to better understand this intriguing observation. The authors already show that S1R depletion results in prM cleavage defects in the medium but it is unclear if this prM is incorporated in viral particles or somehow is independently secreted. qPCR analysis indicates that secreted viral RNA levels are unaffected suggesting that viral particles are being assembled and secreted. Do these secreted viral particles contain less Envelope protein and/or Capsid? This could be determined using western blotting on concentrated medium as the authors performed using prM antibodies. Ideally this would be done after density gradient ultracentrifugation to separate the particles, but I could understand that this falls outside of the scope of the current manuscript.

2. The cleavage defect of prM secreted in the medium is intriguing. If S1R acts as a chaperone, can the cleavage defect be rescued by lowering the temperature during viral assembly and/or can Furin overexpression rescue the cleavage defect?

3. The reduction in detergent solubility is interpreted to reflect misfolding leading to non-soluble protein aggregates. Do the authors see an increase in non-soluble protein aggregates upon S1R KD to more directly show this?

4. The authors shown that upon infection of cells overexpressing HA epitope tagged S1R, pulldown with prM leads to detectable HA-S1R. Did the authors perform the reciprocal pulldown using HA beads and was prM the only protein that co-immunoprecipitated? prM interacts with Envelope and is a chaperone for Envelope.

**********

**Part III – Minor Issues: Editorial and Data Presentation Modifications**

Please use this section for editorial suggestions as well as relatively minor modifications of existing data that would enhance clarity.

Reviewer #1: • What is the impact of S1R depletion on cell growth and viability? Similarly, do the S1R drugs have toxicity?

• Does envelope protein interaction with prM decrease in S1R siRNA treated cells?

• Quantification of Vps should be done per cell rather than per field of view.

Reviewer #2: -S1R seems not to be the official gene name for the protein of interest (i.e. SIGMAR1), which is suggested to be used throughout the manuscript.

-Line 52: Flavivirus is not a virus family. DENV belongs to the Orthoflavivirus genus within the Flaviviridae family. This must be correct.

-Fig 1A-C: What is the impact of S1R knockdown and overexpression on cell viability? This must be shown to rule out that the viral phenotypes are not due to off-target effect or a decreased abundance of virion-producing cells.

-Fig 1C: Western blots showing the knockdown and overexpression efficiency must be shown.

-Fig 1G: Micrographs are shown in duplicate. It is suggested that the authors keep only one image per condition.

-Line 209: In contrast to the conclusion drawn by the authors, NS3 seems to be detected in the control anti-prM IP (HA-mCherry). This signal disappears when S1R is overexpressed. Is this phenotype reproducible and relevant for S1R function?

-Line 156, Fig 2C: The percentage of dsRNA-containing cells is not indicative of the extent of viral RNA replication as concluded by the authors, but instead, simply reflect the proportion of infected cells (i.e., entry or spread depending on the time point post-infection). Instead, the authors should quantify the abundance of dsRNA puncta or the mean fluorescence per cells to be able to draw a conclusion on viral replication.

-Fig 2B: A replication kinetics (1-3 days) following the siRNA transfection of the DENV subgenomic replicon stable cell line would be informative. A negative control disrupting replication (e.g., antiviral drug treatment) should be included. Unless this was missed, no information is provided on how this cell line was generated.

-Is the S1R dependency specific to DENV? If possible, the knockdown replication assays should be repeated with other flaviviruses and an unrelated RNA virus.

-What is the diameter of released viral particles? Are the smaller virions observed inside the cell released?

Reviewer #3: 5. S1R has a role in the formation of cholesterol rich microdomains in the ER and flavivirus prM uses cholesterol for assembly. Can the authors speculate in the discussion whether this may be relevant for the phenotype.

6. In the viral production assays measuring FFU’s there is a wide variance (e.g. in 1B). Even for the NT, there can be a 100-fold difference. Are these experiments biological replicates and are the samples paired?

7. Figure 2D the y-axis states “normalized to NT” this may be a typo.

**********

PLOS authors have the option to publish the peer review history of their article (what does this mean?). If published, this will include your full peer review and any attached files.

If you choose “no”, your identity will remain anonymous but your review may still be made public.

**Do you want your identity to be public for this peer review?**  For information about this choice, including consent withdrawal, please see our Privacy Policy.

Reviewer #1: No

Reviewer #2: No

Reviewer #3: No

[NOTE: If reviewer comments were submitted as an attachment file, they will be attached to this email and accessible via the submission site. Please log into your account, locate the manuscript record, and check for the action link "View Attachments". If this link does not appear, there are no attachment files.]

**Figure resubmission:**

-->While revising your submission, we strongly recommend that you use PLOS’s NAAS tool (https://ngplosjournals.pagemajik.ai/artanalysis) to test your figure files. NAAS can convert your figure files to the TIFF file type and meet basic requirements (such as print size, resolution), or provide you with a report on issues that do not meet our requirements and that NAAS cannot fix.

After uploading your figures to PLOS’s NAAS tool - https://ngplosjournals.pagemajik.ai/artanalysis, NAAS will process the files provided and display the results in the "Uploaded Files" section of the page as the processing is complete. If the uploaded figures meet our requirements (or NAAS is able to fix the files to meet our requirements), the figure will be marked as "fixed" above. If NAAS is unable to fix the files, a red "failed" label will appear above. When NAAS has confirmed that the figure files meet our requirements, please download the file via the download option, and include these NAAS processed figure files when submitting your revised manuscript.

**Reproducibility:**

To enhance the reproducibility of your results, we recommend that authors of applicable studies deposit laboratory protocols in protocols.io, where a protocol can be assigned its own identifier (DOI) such that it can be cited independently in the future. Additionally, PLOS ONE offers an option to publish peer-reviewed clinical study protocols. Read more information on sharing protocols at https://plos.org/protocols?utm_medium=editorial-email&utm_source=authorletters&utm_campaign=protocols

# Author comment: Author response to Decision Letter 1

**DOI:** 10.1371/journal.ppat.1014347.r002

30 Jan 2026

Attachment Submitted filename: Sarkar et al. (Response to Reviewers).docx

# Peer review: Decision Letter 1

**DOI:** 10.1371/journal.ppat.1014347.r003

**Authors:** Alexander Gorbalenya, Ralf Bartenschlager, Alexander Gorbalenya, Ralf Bartenschlager

15 Mar 2026

PPATHOGENS-D-25-02764R1

The Sigma1 ER membrane receptor promotes structural protein folding and genome packaging of Dengue virus

PLOS Pathogens

Dear Dr. Tsai,

Thank you for submitting your manuscript to PLOS Pathogens. After careful consideration, we feel that it has merit but does not fully meet PLOS Pathogens's publication criteria as it currently stands. Therefore, we invite you to submit a revised version of the manuscript that addresses the points raised during the review process.

Please submit your revised manuscript by May 14 2026 11:59PM. If you will need more time than this to complete your revisions, please reply to this message or contact the journal office at plospathogens@plos.org. When you're ready to submit your revision, log on to https://www.editorialmanager.com/ppathogens/ and select the 'Submissions Needing Revision' folder to locate your manuscript file.

Please include the following items when submitting your revised manuscript:

* A letter that responds to each point raised by the editor and reviewer(s). You should upload this letter as a separate file labeled 'Response to Reviewers'. This file does not need to include responses to any formatting updates and technical items listed in the 'Journal Requirements' section below.

* A marked-up copy of your manuscript that highlights changes made to the original version. You should upload this as a separate file labeled 'Revised Manuscript with Track Changes'.

* An unmarked version of your revised paper without tracked changes. You should upload this as a separate file labeled 'Manuscript'.

If you would like to make changes to your financial disclosure, competing interests statement, or data availability statement, please make these updates within the submission form at the time of resubmission. Guidelines for resubmitting your figure files are available below the reviewer comments at the end of this letter.

We look forward to receiving your revised manuscript.

Kind regards,

Ralf Bartenschlager, PhD

Guest Editor

PLOS Pathogens

Alexander Gorbalenya

Section Editor

PLOS Pathogens

Sumita Bhaduri-McIntosh

Editor-in-Chief

PLOS Pathogens

orcid.org/0000-0003-2946-9497

Michael Malim

Editor-in-Chief

PLOS Pathogens

orcid.org/0000-0002-7699-2064

**Additional Editor Comments :**

Dear Dr. Tsai,

Your revised manuscript has now been seen again by the original reviewers. While one reviewer is completely satisfied, two see the strong improvement of your study, yet have some remaining comments. First, both reviewers are not convinced by the prM-IP experiment and the conclusion that you have drawn, including the change of the title. Both reviewers make concrete suggestions how to address this open item. Second, the Western blot of the furin cleavage assay needs to be addressed and I agree to the reviewer’s comment that these samples must be analyzed side-by-side to allow proper comparison. Third, please address the two remaining concerns raised by one of the reviewers.

**Journal Requirements:**

1) We have noticed that you have uploaded Supporting Information files, but you have not included a complete list of legends. Please add a full list of legends for your Supporting Information file"Supplemental Information (Raw Data Fig 1-5+S1).pdf" after the references list.

Note: If the reviewer comments include a recommendation to cite specific previously published works, please review and evaluate these publications to determine whether they are relevant and should be cited. There is no requirement to cite these works unless the editor has indicated otherwise.

**Reviewers' Comments:**

Reviewer's Responses to Questions

**Part I - Summary**

Please use this section to discuss strengths/weaknesses of study, novelty/significance, general execution and scholarship.

Reviewer #1: In the revised manuscript, Sarkar and colleagues have included additional experiments and analyses that strengthen the study and increase confidence in the major conclusions. These new data also provide further insight into the role of SR1 in dengue virus assembly. Overall, the additional work is rigorous and addresses the majority of the concerns raised in the original review.

My remaining concern pertains to the nature and composition of the defective viral particles observed in SR1-depleted cells. The included data convincingly demonstrate a reduction in virion size both intracellularly and in the supernatant. However, although the prM co-immunoprecipitation experiments show decreased association between prM and viral RNA under SR1-depleted conditions, it remains unclear whether this assay is recovering viral particles or instead enriching for secreted prM that is not associated with virions. In particular, the absence of detectable capsid, including in the control condition, suggests that intact virions are unlikely to be efficiently captured by this approach. Additionally, the sucrose gradient analyses indicate enrichment of prM and capsid in distinct fractions following SR1 depletion, with notable accumulation in fraction 5. However, it is not clear whether this material represents the observed defective virions or nonspecific protein aggregates. Additional evidence is needed to establish that the prM immunoprecipitation is isolating virion-associated material and to identify which gradient fraction contains the defective particles. Analysis of viral RNA abundance across the gradient fractions would also help clarify whether the extracellular viral RNA detected in SR1-depleted conditions is secreted within extracellular vesicles, as proposed by the authors (co-fractionation with TSG101), or whether it instead accumulates in higher-density fractions with prM and capsid.

This point does not undermine the overall conclusions of the study, but resolving it would further strengthen the mechanistic interpretation of the defective particle phenotype.

Reviewer #2: In this revised version, Sarkar and colleagues have included new data that convincingly support a role in S1R in assembly. This includes results on intracellular infectivity and morphological analyses of released virions. While they have addressed most of my comments, some questions remain, and newly included controls are not always convincing.

Reviewer #3: The authors have satisfactorily addressed my points.

**********

**Part II – Major Issues: Key Experiments Required for Acceptance**

Please use this section to detail the key new experiments or modifications of existing experiments that should be absolutely required to validate study conclusions.

Generally, there should be no more than 3 such required experiments or major modifications for a "Major Revision" recommendation. If more than 3 experiments are necessary to validate the study conclusions, then you are encouraged to recommend "Reject".

Reviewer #1: (No Response)

Reviewer #2: The authors changed the title of the study with the conclusion that S1R promotes the packaging of dengue virus (DENV) RNA (vRNA) during viral particle assembly. However, this is mostly based on the observation that less vRNA is co-immunoprecipitated with prM from the extracellular medium (Fig 5D). RNA packaging per se was not addressed. It is also counterintuitive that capsid could not be detected in these immunoprecipitates as it normally coats the viral RNA. This raises doubts that this assay detected packaged RNA. To make the proposed claim, the authors should evaluate both capsid and prM association to vRNA inside the cells, that is during the viral assembly process.

The authors did evaluate the impact of a 25 uM treatment with S1Ra and Halo drugs on cell viability (Fig 1G). However, they did not determine their CC50 value as specifically requested. This is of importance to evaluate the selectivity index (CC50/EC50) since the EC50 is between 50 and 76 uM (higher than the concentration tested for viability). Moreover, since S1R regulates assembly, determining the EC50 with FFU assays would be more appropriate than with the % of infection used here.

It is appreciated and relevant that the authors did an additional experiment in which they treated viral particles from S1R-depleted cells with exogenous furin (Fig 5A, middle panel, lane 3). However, the corresponding lane of the western blot is shown independently of the controls which seem to come from a different experiment. This does not allow to compare the different conditions. To appropriately compare the efficacy of prM maturation rescue by furin, the authors must show in the same figure/membrane conditions 1,2 and 3.

Reviewer #3: (No Response)

**********

**Part III – Minor Issues: Editorial and Data Presentation Modifications**

Please use this section for editorial suggestions as well as relatively minor modifications of existing data that would enhance clarity.

Reviewer #1: (No Response)

Reviewer #2: New Fig 5C is interesting. However, to claim that fractions 1-3 contains assembled particles, infectivity assays should be performed with these fractionsOne cannot exclude that fractions 1-3 correspond to extracellular vesicles (since they contain TSG101), free proteins or disassembled particles. In which fractions is the vRNA?

Reviewer #3: (No Response)

**********

PLOS authors have the option to publish the peer review history of their article (what does this mean?). If published, this will include your full peer review and any attached files.

If you choose “no”, your identity will remain anonymous but your review may still be made public.

**Do you want your identity to be public for this peer review?**  For information about this choice, including consent withdrawal, please see our Privacy Policy.

Reviewer #1: No

Reviewer #2: No

Reviewer #3: No

[NOTE: If reviewer comments were submitted as an attachment file, they will be attached to this email and accessible via the submission site. Please log into your account, locate the manuscript record, and check for the action link "View Attachments". If this link does not appear, there are no attachment files.]

**Figure resubmission:**

-->While revising your submission, we strongly recommend that you use PLOS’s NAAS tool (https://ngplosjournals.pagemajik.ai/artanalysis) to test your figure files. NAAS can convert your figure files to the TIFF file type and meet basic requirements (such as print size, resolution), or provide you with a report on issues that do not meet our requirements and that NAAS cannot fix.

After uploading your figures to PLOS’s NAAS tool - https://ngplosjournals.pagemajik.ai/artanalysis, NAAS will process the files provided and display the results in the "Uploaded Files" section of the page as the processing is complete. If the uploaded figures meet our requirements (or NAAS is able to fix the files to meet our requirements), the figure will be marked as "fixed" above. If NAAS is unable to fix the files, a red "failed" label will appear above. When NAAS has confirmed that the figure files meet our requirements, please download the file via the download option, and include these NAAS processed figure files when submitting your revised manuscript.

**Reproducibility:**

To enhance the reproducibility of your results, we recommend that authors of applicable studies deposit laboratory protocols in protocols.io, where a protocol can be assigned its own identifier (DOI) such that it can be cited independently in the future. Additionally, PLOS ONE offers an option to publish peer-reviewed clinical study protocols. Read more information on sharing protocols at https://plos.org/protocols?utm_medium=editorial-email&utm_source=authorletters&utm_campaign=protocols

# Author comment: Author response to Decision Letter 2

**DOI:** 10.1371/journal.ppat.1014347.r004

29 May 2026

Attachment Submitted filename: Sarkar_et_al._(Response_to_Reviewers)_auresp_2.docx

# Editor report: Decision Letter 2

**DOI:** 10.1371/journal.ppat.1014347.r005

**Authors:** Alexander Gorbalenya, Ralf Bartenschlager, Alexander Gorbalenya, Ralf Bartenschlager, Alexander Gorbalenya, Ralf Bartenschlager

3 Jun 2026

Dear Prof. Tsai,

We are pleased to inform you that your manuscript 'The Sigma1 ER membrane receptor promotes structural protein folding and genome packaging of Dengue virus' has been provisionally accepted for publication in PLOS Pathogens.

Before your manuscript can be formally accepted you will need to complete some formatting changes, which you will receive in a follow up email. A member of our team will be in touch with a set of requests.

Please note that your manuscript will not be scheduled for publication until you have made the required changes, so a swift response is appreciated.

IMPORTANT: The editorial review process is now complete. PLOS will only permit corrections to spelling, formatting or significant scientific errors from this point onwards. Requests for major changes, or any which affect the scientific understanding of your work, will cause delays to the publication date of your manuscript.

Should you, your institution's press office or the journal office choose to press release your paper, you will automatically be opted out of early publication. We ask that you notify us now if you or your institution is planning to press release the article. All press must be co-ordinated with PLOS.

Thank you again for supporting Open Access publishing; we are looking forward to publishing your work in PLOS Pathogens.

Best regards,

Ralf Bartenschlager, PhD

Guest Editor

PLOS Pathogens

Alexander Gorbalenya

Section Editor

PLOS Pathogens

Sumita Bhaduri-McIntosh

Editor-in-Chief

PLOS Pathogens

orcid.org/0000-0003-2946-9497

Michael Malim

Editor-in-Chief

PLOS Pathogens

orcid.org/0000-0002-7699-2064

***********************************************************

Reviewer Comments (if any, and for reference):

# Editor report: Acceptance letter

**DOI:** 10.1371/journal.ppat.1014347.r006

**Authors:** Alexander Gorbalenya, Ralf Bartenschlager, Alexander Gorbalenya, Ralf Bartenschlager, Alexander Gorbalenya, Ralf Bartenschlager

Dear Prof. Tsai,

We are delighted to inform you that your manuscript, "The Sigma1 ER membrane receptor promotes structural protein folding and genome packaging of Dengue virus," has been formally accepted for publication in PLOS Pathogens.

We have now passed your article onto the PLOS Production Department who will complete the rest of the pre-publication process. All authors will receive a confirmation email upon publication.

The corresponding author will soon be receiving a typeset proof for review, to ensure errors have not been introduced during production. Please review the PDF proof of your manuscript carefully, as this is the last chance to correct any scientific or type-setting errors. Please note that major changes, or those which affect the scientific understanding of the work, will likely cause delays to the publication date of your manuscript. Note: Proofs for Front Matter articles (Pearls, Reviews, Opinions, etc...) are generated on a different schedule and may not be made available as quickly.

Soon after your final files are uploaded, the early version of your manuscript, if you opted to have an early version of your article, will be published online. The date of the early version will be your article's publication date. The final article will be published to the same URL, and all versions of the paper will be accessible to readers.

For Research Articles, you will receive an invoice from PLOS for your publication fee after your manuscript has reached the completed accept phase. If you receive an email requesting payment before acceptance or for any other service, this may be a phishing scheme. Learn how to identify phishing emails and protect your accounts at https://explore.plos.org/phishing.

Thank you again for supporting open-access publishing; we are looking forward to publishing your work in PLOS Pathogens.

Best regards,

Sumita Bhaduri-McIntosh

Editor-in-Chief

PLOS Pathogens

orcid.org/0000-0003-2946-9497

Michael Malim

Editor-in-Chief

PLOS Pathogens

orcid.org/0000-0002-7699-2064
